# Association of quantitative histopathology measurements with antemortem medial temporal lobe cortical thickness in the Alzheimer’s disease continuum

**DOI:** 10.1007/s00401-024-02789-9

**Published:** 2024-09-03

**Authors:** Amanda E. Denning, Ranjit Ittyerah, Lisa M. Levorse, Niyousha Sadeghpour, Chinmayee Athalye, Eunice Chung, Sadhana Ravikumar, Mengjin Dong, Michael Tran Duong, Yue Li, Ademola Ilesanmi, Lasya P. Sreepada, Philip Sabatini, MaKayla Lowe, Alejandra Bahena, Jamila Zablah, Barbara E. Spencer, Ryohei Watanabe, Boram Kim, Maja Højvang Sørensen, Pulkit Khandelwal, Christopher Brown, Stanislau Hrybouski, Sharon X. Xie, Robin de Flores, John L. Robinson, Theresa Schuck, Daniel T. Ohm, Sanaz Arezoumandan, Sílvia Porta, John A. Detre, Ricardo Insausti, Laura E. M. Wisse, Sandhitsu R. Das, David J. Irwin, Edward B. Lee, David A. Wolk, Paul A. Yushkevich

**Affiliations:** 1 Department of Radiology, University of Pennsylvania, Philadelphia, PA USA; 2 Department of Bioengineering, University of Pennsylvania, Philadelphia, PA USA; 3 Department of Neurology, University of Pennsylvania, Philadelphia, PA USA; 4 Department of Pathology and Laboratory Medicine, University of Pennsylvania, Philadelphia, PA USA; 5 Center for Neurodegenerative Disease Research, Institute On Aging, University of Pennsylvania, Philadelphia, PA USA; 6 Department of Biostatistics, Epidemiology and Informatics, University of Pennsylvania, Philadelphia, PA USA; 7 UMR-S U1237, PhIND “Physiopathology and Imaging of Neurological Disorders”, Institut Blood and Brain @ Caen-Normandie, INSERM, Caen-Normandie University, GIP Cyceron, Caen, France; 8 Human Neuroanatomy Lab, University of Castilla La Mancha, Albacete, Spain; 9 Department of Clinical Sciences Lund, Lund University, Lund, Sweden

**Keywords:** Alzheimer’s disease, Quantitative neuropathology, Tau, TDP-43

## Abstract

The medial temporal lobe (MTL) is a hotspot for neuropathology, and measurements of MTL atrophy are often used as a biomarker for cognitive decline associated with neurodegenerative disease. Due to the aggregation of multiple proteinopathies in this region, the specific relationship of MTL atrophy to distinct neuropathologies is not well understood. Here, we develop two quantitative algorithms using deep learning to measure phosphorylated tau (p-tau) and TDP-43 (pTDP-43) pathology, which are both known to accumulate in the MTL and are associated with MTL neurodegeneration. We focus on these pathologies in the context of Alzheimer’s disease (AD) and limbic predominant age-related TDP-43 encephalopathy (LATE) and apply our deep learning algorithms to distinct histology sections, on which MTL subregions were digitally annotated. We demonstrate that both quantitative pathology measures show high agreement with expert visual ratings of pathology and discriminate well between pathology stages. In 140 cases with antemortem MR imaging, we compare the association of semi-quantitative and quantitative postmortem measures of these pathologies in the hippocampus with in vivo structural measures of the MTL and its subregions. We find widespread associations of p-tau pathology with MTL subregional structural measures, whereas pTDP-43 pathology had more limited associations with the hippocampus and entorhinal cortex. Quantitative measurements of p-tau pathology resulted in a significantly better model of antemortem structural measures than semi-quantitative ratings and showed strong associations with cortical thickness and volume. By providing a more granular measure of pathology, the quantitative p-tau measures also showed a significant negative association with structure in a severe AD subgroup where semi-quantitative ratings displayed a ceiling effect. Our findings demonstrate the advantages of using quantitative neuropathology to understand the relationship of pathology to structure, particularly for p-tau, and motivate the use of quantitative pathology measurements in future studies.

**Supplementary Information:**

The online version contains supplementary material available at 10.1007/s00401-024-02789-9.

## Introduction

The medial temporal lobe (MTL) is an epicenter of pathology and atrophy in multiple neurodegenerative diseases. Measurements of thickness and volume of the MTL and its subregions derived from in vivo magnetic resonance imaging (MRI) are associated with cognitive decline and can serve as effective biomarkers of disease progression [40, 66, 87, 97, 101]. Advancements in MRI and image processing techniques have enabled in vivo imaging and quantitative analysis of the anatomically and functionally distinct subfields/subregions that make up the hippocampus and MTL [36, 74, 101, 104]. Volume and thickness measurements of these subregions have been used in aging and neurodegenerative disease studies examining longitudinal subregional atrophy patterns, functional connectivity, and positron emission tomography (PET) imaging of amyloid and tau pathology [3, 13, 34, 76, 100].

However, multiple neurodegenerative pathologies contribute to neurodegeneration in the MTL, often concurrently, including those involved in Alzheimer’s disease (AD), Lewy Body Disease (LBD), hippocampal sclerosis (HS), and limbic predominant age-related TDP-43 encephalopathy (LATE) [27, 43, 55, 77–79, 83, 96, 102]. Therefore, while measurements of MTL volume and cortical thickness can reflect neurodegeneration associated with neurodegenerative disease in general, the specificity of these patterns as downstream effects of distinct neuropathologies is not well understood. With the recent availability of disease-modifying treatments for AD [23, 82], there is increased need to measure the likelihood of non-AD pathology in individual patients, as this may impact decisions to start treatment as well as the degree of treatment-response. Targeted in vivo biomarkers for non-AD neuropathologies are currently lacking. Fluid biomarkers for alpha-synuclein [30] and TDP-43 [12] are promising, but will require further validation. Biomarkers that use MRI, PET, or both, to infer the presence of TDP-43 [4, 9, 27, 59] and alpha-synuclein [17, 21, 48, 49, 58, 62] have been proposed, but still have only limited validation. Therefore, understanding how distinct neurodegenerative pathologies impact specific subregions of the MTL may help inform detection of AD and other neuropathologies in vivo and monitoring of patterns of pathology-specific downstream neurodegeneration in clinical research.

Postmortem work combining neuroimaging with pathology measurements is frequently used to study these associations. Prior studies of pathology-atrophy associations in the MTL, using either ex vivo or antemortem MRI, have shown subtle but somewhat inconsistent differences in the relationships between different pathologies and MTL subregional cortical thickness [19, 31, 53, 77, 96]. Such studies have primarily utilized staging systems or semi-quantitative ordinal ratings of pathology (e.g., none, mild, moderate, severe) derived at the time of neuropathological examination as measures of pathological burden. Staging methods, such as the Braak staging system for tau pathology in AD [6], are global descriptors of topographical spread and pathology progression through the brain, but offer only limited information on pathology burden in specific regions. Semi-quantitative visual ratings of pathology in individual brain regions provide an alternative, but are subjective and difficult to harmonize across studies, and cannot capture the variation of pathology burden within a given rating. Recent advancements in histological image analysis have allowed for the development of automated tools to quantify pathology burden from whole-slide digital images. A number of approaches for quantifying amyloid-beta plaques and tau neurofibrillary tangles (NFTs) in AD show high accuracy when compared to manual counts [71, 81, 84, 90, 99]; others have also been developed to quantify TDP-43 pathology [56]. The limited number of studies that used quantitative pathology measures in neurodegenerative disease studies found correlations with clinical features such as cognitive testing and duration of illness [16, 24, 47, 91] that were not found when using classic staging schemes or semi-quantitative ordinal ratings [37, 61, 92].

Here, we aim to characterize the patterns of MTL neurodegeneration, as measured by antemortem MRI, associated with two proteinopathies that profoundly impact MTL atrophy: phosphorylated tau (p-tau) pathology in AD neuropathological change (ADNC), and phosphorylated TDP-43 (pTDP-43) pathology in LATE neuropathological change (LATE-NC). Both pathologies follow stereotypical progression patterns within the MTL [5, 6, 45, 69] and are associated with MTL atrophy and cognitive decline [3, 18, 29, 47, 70, 85]. According to Braak & Braak, tau pathology in AD begins in the transentorhinal region before spreading to hippocampus and eventually the isocortex [5, 6]. Early stages of LATE-NC involve TDP-43 pathology in the amygdala before progression to the hippocampus and finally neocortex [45, 69]. Tau and TDP-43 also have high rates of co-occurrence in the MTL, and their co-occurrence is linked to higher odds of dementia and more rapid disease progression [39, 43, 50]. Thus, disentangling the relationship of MTL atrophy with these two pathologies has important implications for patient care and clinical trials. Here, we evaluate the association of p-tau and pTDP-43 pathology with structural measures of MTL subregions, using antemortem neuroimaging of the MTL to promote generalizability of our findings to in vivo imaging biomarkers. Based on previous studies of these pathologies and structure, we hypothesize that tau and TDP-43 pathology will be linked to different patterns of MTL atrophy, with greatest p-tau associations in the transentorhinal region (medial BA35, lateral ERC) and throughout the long axis of the hippocampus [3, 77, 85, 93, 96] while pTDP-43 will be more tightly linked to atrophy in the anterior hippocampus and ERC [2, 27, 77, 95]. An additional aim was to compare the utility of quantitative measures of p-tau and pTDP-43 burden extracted from digital histology images using a machine learning approach [22, 103] to semi-quantitative ratings assigned by neuropathologists in modeling structure. We hypothesize that quantitative measures will be more strongly associated with MTL atrophy compared to semi-quantitative ratings, particularly when restricting the analysis to individuals in the most severe stages of disease, where we expect ordinal semi-quantitative ratings to experience a “ceiling effect,” limiting their ability to explain variation in MTL atrophy. We test these hypotheses in a large cohort of brain donors (*n* = 140) with antemortem MRI and postmortem neuropathology examination.

## Materials and methods

Deceased research study participants from the Penn Alzheimer’s Disease Research Center and Penn Frontotemporal Dementia Center who underwent antemortem MRI at Penn, donated their brain for research, and underwent autopsy at the Penn Center for Neurodegenerative Disease Research (CNDR) were included in this study. Patients whose neuropathological diagnoses included non-AD tauopathies, frontotemporal lobar degeneration (FTLD), or amyotrophic lateral sclerosis (ALS) were excluded, resulting in patients with diagnoses including ADNC, primary age-related tauopathy (PART), LBD, cerebrovascular disease (CVD), cerebral amyloid angiopathy (CAA), HS, and LATE-NC. We refer to this cohort composition as the “Alzheimer’s continuum” since it includes the full range of tangle and plaque pathology as well as concomitant pathologies that are common in ADNC.

### Antemortem MRI acquisition and analysis

All patients underwent T1-weighted antemortem imaging during life at the University of Pennsylvania. All imaging procedures during life were performed with informed consent in accordance with Penn Institutional Review Board Guidelines and the Declaration of Helsinki. These scans took place from 1999 to 2021 at 1.5 T or 3 T magnetic field strengths. Magnetic field strength, scanner type, and acquisition parameters (i.e., repetition time, echo time, flip angle, and voxel output size) varied over the course of the study; additional information on these values can be found in the Supplementary Information. Scans were processed using T1-ASHS, a previously validated pipeline for automatic segmentation of MTL subregions [101]. Segmented regions include anterior hippocampus (AH), posterior hippocampus (PH), entorhinal cortex (ERC), Brodmann areas 35 (BA35) and 36 (BA36), which are parts of the perirhinal cortex, and parahippocampal cortex (PHC), as well as the surrounding white matter, sulci, and meninges. A surface-based pipeline CRASHS [105] based on the Nighres package [35] was used to extract the mid-surface between the gray/white matter and gray matter/CSF boundaries, inflate this surface to achieve a simplified, smoothed shape, and perform surface-based registration between inflated surfaces and a surface template. Gray matter thickness was computed for each participant and mapped into the template, allowing thickness measures from all participants to be analyzed at each point of the template. Additionally, summary measures for each subregion were computed, following [101]: median thickness measurements for the more “sheet-like” MTL extrahippocampal subregions (ERC, BA35, BA36, and PHC) and total volume for AH and PH. All segmentations were visually inspected (see [94] for the protocol); poor-quality scans or failed segmentations were excluded from analysis on a per hemisphere basis. For all participants, the scan closest to death with the most hemispheres passing segmentation quality control was utilized. Only scans within 10 years before death were included in the final imaging analyses.

### Neuropathological staging (semi-quantitative measures)

For each donor, one hemisphere underwent extensive pathology processing at the Penn CNDR, as described previously [88]. The hemisphere side to be evaluated was generally randomly selected. Tissue was embedded in paraffin blocks and cut into 6 µm sections for immunohistochemistry using primary antibodies of NAB228 (monoclonal antibody [mAb], 1:30,000*,* generated in the CNDR) to detect amyloid deposits and for Thal staging, p-tau PHF1 (mAb, 1:3000, a gift from Dr. Peter Davies) to detect p-tau deposits and for Braak staging, 1D3 (mAb, 1:300*,* a gift from Dr. Manuela Neumann and Dr. Elisabeth Kremmer) to detect pTDP-43 deposits, and Syn303 (mAb, 1:30,000*,* generated in the CNDR) to detect the presence of pathological ɑ-synuclein. Ratings of neuropathology in each region, as well as neuronal loss, were scored as absent (0), rare (0.5), mild (1), moderate (2), and severe (3). The semi-quantitative ratings of pathology are consensus-based ratings; each case is examined by an initial rater and then reviewed with an expert neuropathologist. Semi-quantitative evaluation of pathology is standard practice and has been previously validated in multicenter studies [64] and at Penn [38]. Neuropathologists also applied the 2012 NIA-AA criteria for global neuropathological Thal, Braak, and Consortium to Establish a Registry for Alzheimer’s Disease (CERAD) staging, as well as neuropathological diagnoses [65]. LATE-NC was staged according to established criteria [69] using semi-quantitative neuropathology ratings of pTDP-43 pathology. Ordinal ratings of pathology in the amygdala were considered for stage 1, in the dentate gyrus, cornu ammonis 1 (CA1) and subiculum, and entorhinal cortex for stage 2, and in the middle frontal gyrus for stage 3. Entorhinal cortex ratings were most frequently at the level of the hippocampal body though occasionally at the amygdala, and as such were used to determine LATE-NC stage 2 pathology. A rating of 1 (mild) or greater in these regions was considered positive. Finally, semi-quantitative ratings of pathology in three MTL regions (entorhinal cortex, CA1/subiculum, and dentate gyrus) were averaged to create semi-quantitative ratings of p-tau and pTDP-43 pathology in the MTL; higher values of this average correspond to more pathology. This process was repeated for ratings of neuronal loss. These averages are referred to as the semi-quantitative MTL p-tau (or pTDP-43 or neuronal loss) rating throughout.

### Quantitative pathology methods

Histology slides previously used for neuropathological staging were digitally scanned on the Huron LT120 optical scanner at 0.4 × 0.4 µm resolution or on the 3DHistech Panoramic P250 scanner at 0.24 µm × 0.24 µm resolution and transferred to an open-source web application, “PICSL histology annotation server (PHAS)” (https://github.com/pyushkevich/histoannot), for viewing and annotating. A deep learning based weakly supervised learning (WSL) algorithm, *WildCat* [22], was adapted to the task of labeling pathological inclusions in histology slides, similar to earlier work [103]. *WildCat* examines image patches of dimension 224 × 224 pixels (sampled from digital histology images resampled to 0.4 µm × 0.4 µm resolution) and assigns each patch a class label (e.g., tangles, threads, background, or other for p-tau). *WildCat* also generates a spatial attention heat map that indicates the location and intensity of features driving the classification decision. Training data for *WildCat* were generated by human raters who placed thousands of rectangular boxes around examples of different classes of pathological inclusions. In previous work, we trained *WildCat* to detect p-tau threads, p-tau tangles, normal-appearing tissue, and slide background on 50 µm thick AT8-stained slides and showed that the attention maps generated by the algorithm on novel slides unseen during training provide a rough pixel-level segmentation of tangles. Summary measures of tangle burden obtained by integrating these attention maps over regions of interest were strongly correlated with manual tangle counts in these regions, as well as with an expert’s semi-quantitative ratings [103].

In the current work, two new classifiers were trained (i) for labeling p-tau inclusions in PHF1-stained 6 µm thick sections and (ii) for labeling pTDP-43 inclusions in 1D3-stained 6 µm thick sections, as detailed below.

### P-tau pathology training data

For p-tau, training data for *WildCat* was generated by 22 raters over the course of a single day event. Raters received training from and were supervised by neuropathology experts, who also participated in annotating training data. Raters had access to the full-resolution whole slide images of p-tau PHF1-stained slides on the PHAS system and the patients’ neuropathological diagnoses and global pathological burden ratings. Using the PHAS system, raters were asked to draw boxes to mark a variety of neuronal, dendritic, and glial p-tau inclusions as well as normal appearing tissue, artifacts, and background. Criteria for annotation were based on the training from [103], with additional information from [11, 25, 26, 54, 65, 89] and input from the expert neuropathologists. Sections from 37 specimens spanning different neuropathological diagnoses and stages were included for training. Further details on the training data and criteria are given in the Supplementary Information.

Training labels were grouped into four broader classes of background (including healthy neurons, tissue, non-tissue background, artifact, stain background, and dirt), tangles (including NFTs, non-pyramidal tangles, and pre-tangles), threads (including gray matter threads, neuritic plaques, tangle associated neuritic clusters, axonal threads, and grains), and other (including astrocytic p-tau, coiled bodies, ballooned neurons, and globular glial tauopathies) for training of *WildCat*. A total of 10,304 training examples were generated (Supplemental Table 1).

### pTDP-43 pathology training data

For pTDP-43, training data for *WildCat* were generated by 7 raters over the course of three months. Neuropathology experts trained and supervised these raters, who had access to the full-resolution whole slide images of pTDP-43 1D3-stained slides but were blinded to any neuropathology ratings or diagnoses. Raters were asked to use boxes to mark a variety of neuronal, neuritic, and glial pTDP-43 pathologies, as well as normal appearing tissue. Criteria for the training were created through discussion with expert neuropathologists and review of current research [7, 8, 11, 44, 57, 68, 72, 73, 86]. Sections from 65 specimens across neuropathological diagnoses and stages were included for training (more information on the specimens and annotation criteria are provided in the Supplementary Information).

Training labels were grouped into classes of background (including healthy neurons, background, tissue, dirt, artifact, and vacuoles), neuronal/glial (round neuronal cytoplasmic inclusions (NCIs), ring-like NCIs, neuronal intranuclear inclusions (NIIs), and white matter oligodendroglial inclusions), neuritic (short dystrophic neurites, long dystrophic neurites, white matter threads, and grains), and non-specific staining (including granulovacuolar degeneration, neuromelanin, and other non-specific staining). A total of 12,489 training examples were generated (Supplemental Table 2).

### *WildCat* training

**Fig. 1:**
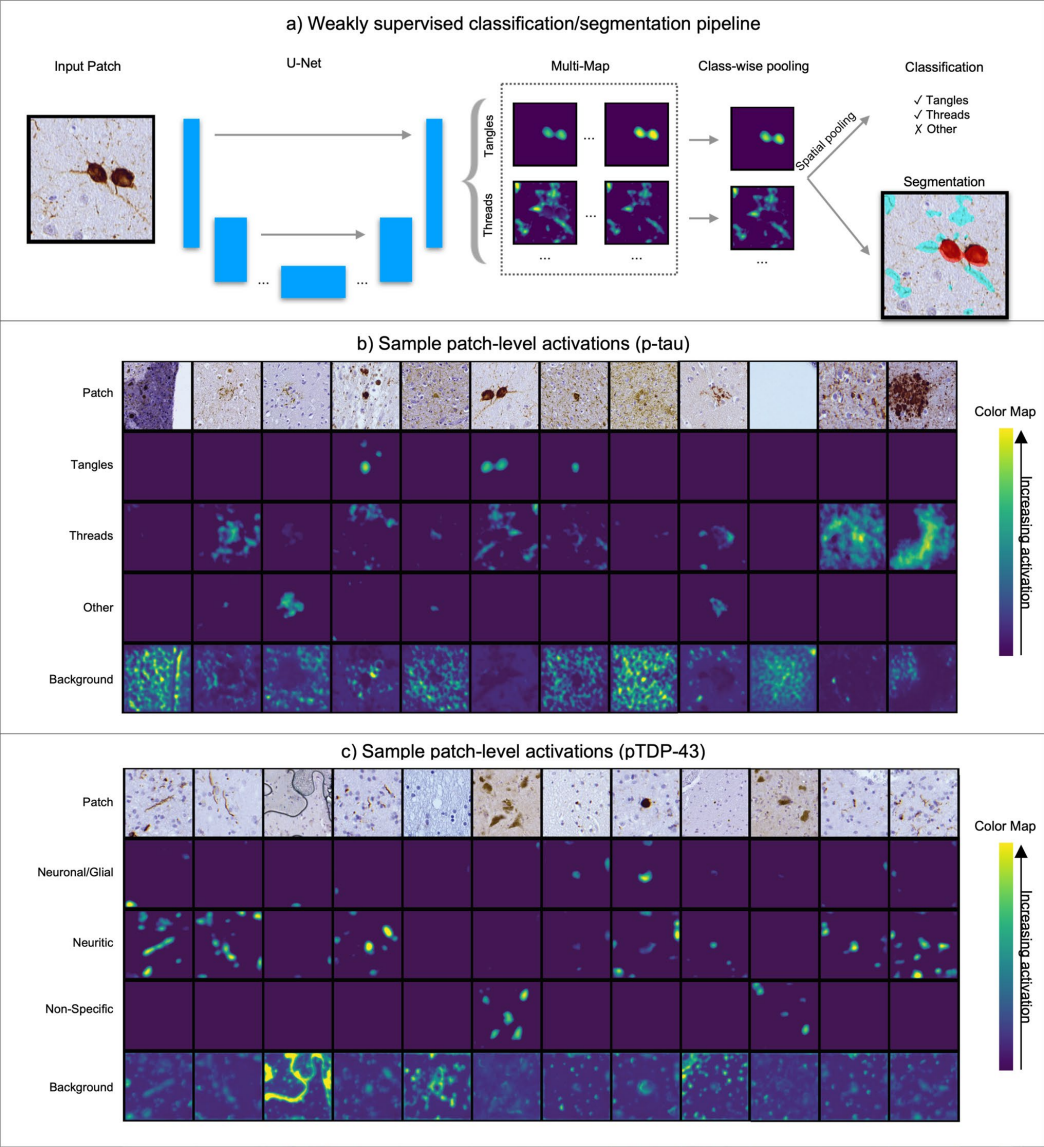
WildCat pipeline and sample activation maps. **a** Pipeline for WildCat classification of p-tau inclusions. The pipeline is the same for the WildCat trained for pTDP-43 inclusions, replacing tangles and threads with neuronal/glial and neuritic pTDP-43. **b** Sample patch level activations for classification of p-tau inclusions. Shown are example patches and heatmaps showing the activation for each class (tangles, threads, other, and background). **c** Sample patch level activations for classification of pTDP-43 inclusions. Shown are example patches and heatmaps showing the activation for each class (neuronal/glial pTDP-43, neuritic pTDP-43, non-specific staining, and background). Green areas in (**b**) and (**c**) correspond to areas of higher activation

To evaluate the accuracy of *WildCat* patch-level classification, five-fold cross-validation experiments were conducted. Annotated specimens for each pathology were randomly split into five subsets, and, in each cross-validation experiment, four subsets were used for *WildCat* training and validation (a small validation subset is used to determine stopping criteria to avoid overfitting) and the remaining subset was used as the test set. For each cross-validation experiment, we quantified the sensitivity and specificity of the labels predicted by the corresponding *WildCat* model. Finally, for each pathology, a single *WildCat* model was trained using patches from all the annotated specimens. These models were used in subsequent analyses. A visualization of the *WildCat* pipeline and example patches for both p-tau and pTDP-43 are shown in Fig. 1.

### Anatomical sampling regions of interest

To imbue quantitative measurements of pathology with anatomic specificity, sampling boxes in anatomical regions of interest (ROIs) were annotated on PHF1 and 1D3 digital histology images from all cases with eligible neuropathological diagnoses and any antemortem imaging. Given the large amount of effort that such annotation requires, only histology sections including the hippocampus were annotated in the current paper, as it is an early site of both p-tau and pTDP-43 pathology. The sampling boxes were placed using the PHAS system. Anatomical regions labeled included CA1, CA2, and CA3, the granular cell layer of the dentate gyrus, the hilus of the dentate gyrus, a transition area between CA1 and subiculum, a transition area between subiculum and presubiculum, three areas within the entorhinal cortex corresponding to the areas most medial, central, and lateral on the slide, BA35, and BA36; see the Supplementary Information for details on the annotation protocol.

**Fig. 2:**
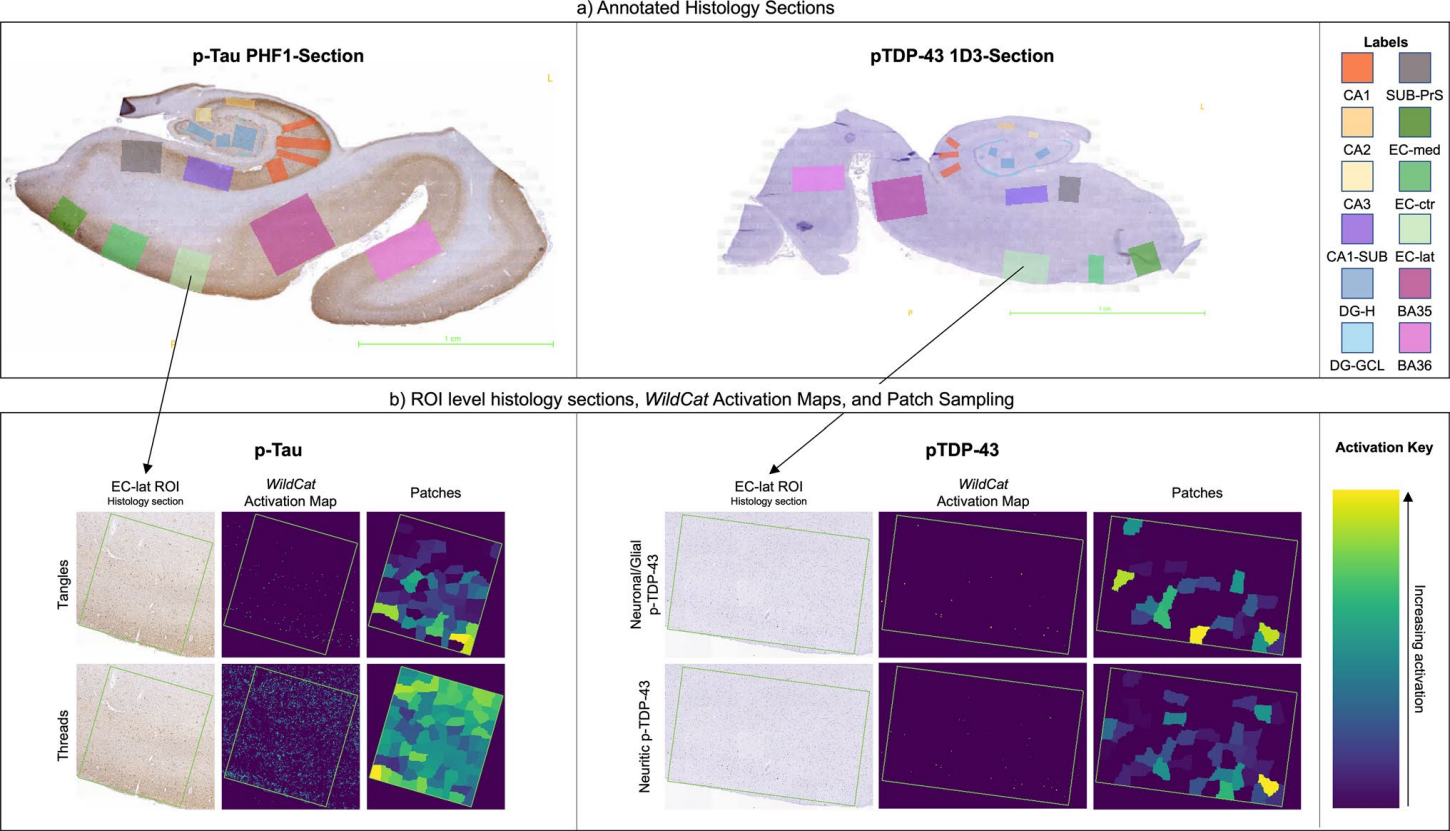
Examples of annotated hippocampus histology sections and corresponding *WildCat* activation maps. **a** A p-tau-stained section (left) and a pTDP-43-stained section (right) with sampling regions of interest annotated. The p-tau section is from a case with high ADNC; the pTDP-43 section is from a case with LATE-NC stage 2. The colors of each label are given to the right. **b** Shown is the EC-lat (lateral area of entorhinal cortex) region of interest labeled from the same section, the *WildCat* activation heat map for tangles and threads (p-tau) or neuronal/glial and neuritic pTDP-43, and the same ROI broken into approximately equal size contiguous segments from which the average activation measure is taken. These segments are obtained using the METIS graph partitioning algorithm [51]. Yellow and green areas indicate areas of higher activation. *CA* cornu ammonis, *CA1-SUB* cornu ammonis 1/subiculum, *DG-H* hilus of the dentate gyrus, *DG-GCL* granular cell layer of the dentate gyrus, *SUB-PrS* subiculum/presubiculum, *EC-med* medial portion of the entorhinal cortex visible on the section, *EC-ctr* central portion of entorhinal cortex visible on the section, *EC-lat* lateral portion of entorhinal cortex visible on the section, *BA* Brodmann area

Annotation yielded a total of 1,585 regions of interest annotated across 200 PHF1 slides. To reduce manual effort, piecewise diffeomorphic deformable registration between hippocampal PHF1 and 1D3 slides was performed for each individual using the multi-stain registration algorithm in [73] and ROIs were mapped onto the corresponding 1D3 slides. ROIs were manually corrected for registration errors and to avoid areas of significant tissue damage. When registration failed or was not possible (e.g., p-tau and pTDP-43 slides in different blocks, or only one stain available), the pTDP-43 slide was manually annotated following the same protocol. Registration and manual annotation resulted in 1,576 regions of interest annotated across 194 1D3-stained slides. Examples of annotated p-tau and pTDP-43 sections are shown in Fig. 2.

### Extraction and evaluation of ROI-level quantitative pathology measures

Heat maps of pathology burden (tangles and threads for p-tau; neuronal/glial and neuritic for pTDP-43) were generated for each sampling box by applying the corresponding trained *WildCat* model. From each of these heat maps, a summary measure of pathology burden was generated by breaking each sampling box into small (~ 200 × 200 µm) segments (examples of these segments are shown in Fig. 2), averaging the heat map over each segment, and taking a summary statistic (e.g., 90th percentile, mean) across all segments. To validate these box-level summary measures, hippocampal sections from cases with passing quality antemortem MRI not included in the model training were stratified by the semi-quantitative MTL ratings of p-tau, and 64 sampling boxes were randomly selected for validation, and likewise for pTDP-43. An expert rater (p-tau: SA, pTDP-43: JR) examined each sampling box and assigned separate semi-quantitative ratings (0–3) of tangle and thread pathology for p-tau or neuronal/glial and neuritic pathology for pTDP-43. As the 64 sampling boxes for pTDP-43 showed a skew towards less pathology, 29 additional sampling boxes were selected from the high MTL pTDP-43 strata to ensure the full range of pathology was captured for validation. Quantitative measures for each pathology were generated for each sampling box in this validation dataset. The agreement between the experts’ ratings and the corresponding box-level quantitative measures generated from aggregating the segment-level averages was evaluated statistically via receiver operating characteristic (ROC) curve analysis and Mann–Whitney tests. For each of the four types of pathology measures, we tested the ability of the box-level summary quantitative ratings to discriminate between adjacent semi-quantitative categories. The analysis was repeated using different summary statistics (mean, median, maximum, 25th, 75th, 90th, 95th, and 99th quantiles) to determine the summary statistic that most consistently agreed with experts’ ratings across all four pathology categories. Additional details can be found in the Supplementary Information.

For subsequent analyses, quantitative pathology measures were calculated for each anatomical ROI rather than each sampling box since most anatomical ROIs were sampled using several boxes. To do this, segment-level heat map averages for each anatomical ROI were pooled across the multiple sampling boxes, and the best-performing quantile statistic was applied. This yielded quantitative measures of tangle and thread burden for each anatomical ROI in each p-tau section, and, similarly, measures of neuronal/glial and neuritic burden for each anatomical ROI in each pTDP-43 section.

### Hippocampus-level summary quantitative pathology measures

Summary measures of hippocampal pathology burden were generated for each participant by averaging the ROI-level pathology measures across CA1, CA2, CA3, CA1/subiculum, subiculum/presubiculum, the hilus of the dentate gyrus, and the granular cell layer of the dentate gyrus. We included these regions and not others in the average because they could be commonly sampled across our dataset (i.e., not frequently torn or damaged) and because they can be identified consistently along the hippocampal longitudinal axis. Conversely, ROIs from the MTL cortex were annotated, but not included in the average, because there was significant variation in CNDR slides as to where along the longitudinal axis the hippocampus was sectioned (body vs. head). Without a reference ex vivo MRI or serial histology, it is difficult to estimate the exact location of a histology slide along the longitudinal axis. This variation makes it difficult to assess whether the MTL cortex present on each slide contains entorhinal cortex and BA35/36, as expected on more anterior sections, or parahippocampal cortex, as expected on more posterior sections. A summary hippocampal pathology measure was not computed for a participant if fewer than three anatomical ROIs from the list above could be annotated; ROI measurements available in both dentate gyrus subregions were considered as only one region towards this count (though their measures were calculated separately, and both included in the average).

To make the scale of the hippocampus-level summary quantitative pathology measures more meaningful, we applied a linear transformation to the hippocampal quantitative summary measures based on the linear fit between the untransformed quantitative measures and the corresponding semi-quantitative MTL p-tau or pTDP-43 ratings. This transformation puts the summary quantitative measures roughly in a range between 0 and 3, with 0 corresponding to little or no burden and 3 corresponding to severe burden. This simple linear transformation has no impact on the associations with MRI-derived measures and is only performed to improve the interpretability of these measures. We refer to this transformed average as the “summary measure” for each inclusion type (e.g., p-tau tangles) throughout.

### Statistics

All analyses were performed in R version 4.3.0. Validation of the quantitative pathology measurements in individual sampling regions was evaluated with one-sided Mann–Whitney area under the curve (AUC) tests between adjacent expert ratings. Additional validation of the quantitative summary measures included Spearman correlations of the summary measure with the average MTL p-tau and pTDP-43 ratings and average neuronal loss rating in the MTL. Quantitative pathology summary scores were also compared to Braak (p-tau) and LATE-NC (pTDP-43) staging through one-sided Mann–Whitney AUC tests. These pathology analyses included all cases with p-tau or pTDP-43 quantitative summary pathology measurements regardless of MRI or segmentation quality.

For the pathology and imaging analyses, cases with quantitative p-tau and pTDP-43 summary measures were included if the antemortem MRI passed segmentation and image quality control in the hemisphere ipsilateral to the one used for neuropathology. This group is referred to as the “whole imaging cohort.” To examine the relationships between MRI-derived measures and quantitative pathology summary measures in cases with more severe pathology burden, where we hypothesized that we would observe “ceiling effects” with semi-quantitative measures, we defined the advanced ADNC subgroup as participants with Braak stages V or VI. As only a small number of individuals had LATE-NC stage 2 or 3, we defined the LATE-NC subgroup as participants with LATE-NC stage 1 or greater. In the overall cohort and these two subgroups, linear models were fit for each MTL subregion using T1-ASHS derived volume (AH/PH) or median thickness (ERC/BA35/BA36/PHC) as the dependent variable and p-tau and pTDP-43 summary pathology measures as the independent variables.

To determine which combination of pathology predictors to analyze in these models (i.e., semi-quantitative or quantitative measures for both p-tau and pTDP-43), we performed post-hoc analyses to evaluate the differences in the strength of the association between quantitative summary pathology measures and semi-quantitative ratings with structure. For p-tau, we generated two linear models for each imaging ROI; a quantitative model including the tangles and threads summary measures and covariates of age, antemortem interval (time from MRI to date of death), sex, MRI field strength, and the semi-quantitative MTL pTDP-43 rating, as well as intracranial volume (ICV) for the 2 regions with volume computed (AH/PH). The semi-quantitative model included the MTL p-tau rating and the same covariates. These models included all patients with ipsilateral imaging and quantitative p-tau summary measures available. We computed the differences between these two models in adjusted *R*^2^, prediction error, Akaike’s information criterion with correction for small sample sizes (AICc), and Bayesian information criterion (BIC). Prediction error was calculated as the sum of the absolute value (L1 norm) of the residuals for each model. A difference of greater than 2 in BIC was considered positive evidence of a better model fit, greater than 6 as strong evidence, and greater than 10 as very strong evidence [75]. These tests were also performed for pTDP-43 with pathology variables of hippocampal neuronal/glial and neuritic pTDP-43 summary measures in the quantitative model, the MTL pTDP-43 rating in the semi-quantitative model, and the semi-quantitative MTL p-tau rating as a covariate for both.

Based on these model comparisons, we selected the best pathology measures (either quantitative or semi-quantitative measures for p-tau and pTDP-43) to analyze in one linear model for each pathology to structure analysis in both the whole imaging cohort and the two subgroups (advanced ADNC and LATE-NC). Cases were included if the antemortem MRI passed segmentation quality control in the hemisphere ipsilateral to the one used for neuropathology and they had the required pathology measurements. Each model included age at death, sex, MRI field strength, and antemortem interval as nuisance covariates. ICV, computed using [101], was included as an additional covariate for AH and PH volume, since hippocampal volume measures, but not cortical thickness measures, are expected to correlate with head size [1, 80]. For each fitted model, the standardized β coefficients, t-statistics, and one-sided *p*-values for the contrasts of interest (e.g., association between tangle summary measure and ERC median thickness) were computed and reported. One-sided tests examined only negative associations as we expected all pathology to be negatively associated with neurodegeneration. We did not correct for multiple comparisons due to strong a priori hypotheses that structure and pathology should be negatively associated. However, results that would survive Bonferroni correction at *P* < 8.33e-03 (0.05 / 6 regions examined) are also indicated in the table.

Finally, three-dimensional surface maps were generated comparing the measure of gray matter thickness for each point in the MTL to the given pathology measures. These models were analogous to the ROI-level linear models, but with additional correction for multiple comparison using the cluster-level inference approach based on permutation testing [32], as implemented in the **meshglm** program (https://github.com/pyushkevich/cmrep). Specifically, after mapping all thickness values to a common geometrical template, fitting a linear model at each point in the template, and computing a *t*-statistic map for the contrast of interest at each point, we extracted contiguous clusters based on the threshold *t *> 2.0 and recorded the surface area of these clusters. We then computed the null distribution of maximum cluster surface area under random permutation (*n* = 10,000) of data labels, following the two-step Freedman-Lane procedure [28]. For each cluster, the one-sided corrected *p* value was computed as the proportion of permutation experiments in which a cluster of larger surface area was extracted.

## Results

### Demographics

229 brain donors met our inclusion criteria based on neuropathological diagnoses along the AD continuum and antemortem imaging. One autopsy was excluded due to a very young age at death (< 35 years). After excluding cases with fewer than three annotated anatomical ROIs, 196 cases with PHF1-stained p-tau sections were available in the p-tau pathology analyses (“Tau Pathology Cohort”) and 190 cases for pTDP-43 analyses (“TDP-43 Pathology Cohort”). For the imaging analyses, 163 patients had a usable antemortem T1-weighted MRI within 10 years prior to death that passed segmentation quality control on the ipsilateral hemisphere that was sectioned for neuropathology (“Imaging Cohort”); this number includes patients regardless of quantitative pathology availability. Demographics for these three groups are given in Table 1.

**Table 1:**
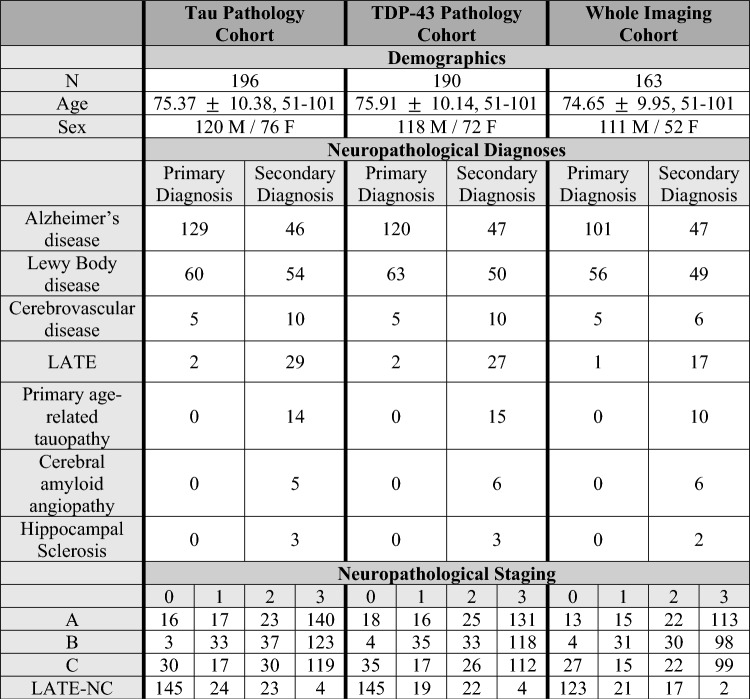
Demographics given for cases with quantitative p-tau data, quantitative pTDP-43 data, and ipsilateral MRI and segmentation passing QC

Cases with quantitative p-tau summary measures were included in the p-tau pathology-only analyses, and likewise for pTDP-43. The imaging cohort includes cases with usable antemortem MRI within 10 years prior to death and passing quality segmentation on the ipsilateral hemisphere to the one sectioned for neuropathology, regardless of the availability of quantitative pathology measures. *A* Amyloid, *B* Braak, *C* CERAD, *LATE-NC* Limbic predominant age-related TDP-43 encephalopathy neuropathologic change

### Quantitative pathology validation

The average accuracy of five-fold patch-level cross-validation of WildCat was 85.2% for p-tau tangles and 73.5% for p-tau threads. Accuracy was higher for pTDP-43: 92.7% for neuronal/glial and 95.3% for neuritic inclusions (Table 2). Cross-validation results for each kind of inclusion within each of these classes are shown in Supplementary Tables 1 (p-tau) and 2 (pTDP-43). Low accuracy in the threads measurement is mostly attributable to misclassification of neuritic plaques (often as tangles or other) and axonal threads (most often as background), the latter of which is expected to be rare in our dataset as our sampling regions are placed only in gray matter. Astrocytic p-tau was also commonly misclassified in cross-validation as threads (~ 29%) rather than the correct “other” class.

**Table 2:**
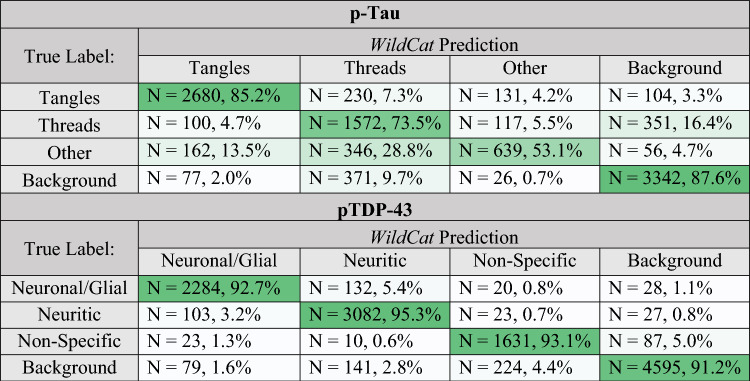
Five-fold cross-validation for *WildCat* training, grouped by class, for p-tau and pTDP-43 measurements

Given is the number of patches (N) in each *WildCat* prediction class stratified by the true label annotated during training. The percentages evaluate the number of patches predicted in each class by *WildCat* within a given true label. Darker green colors indicate higher percentages

**Fig. 3:**
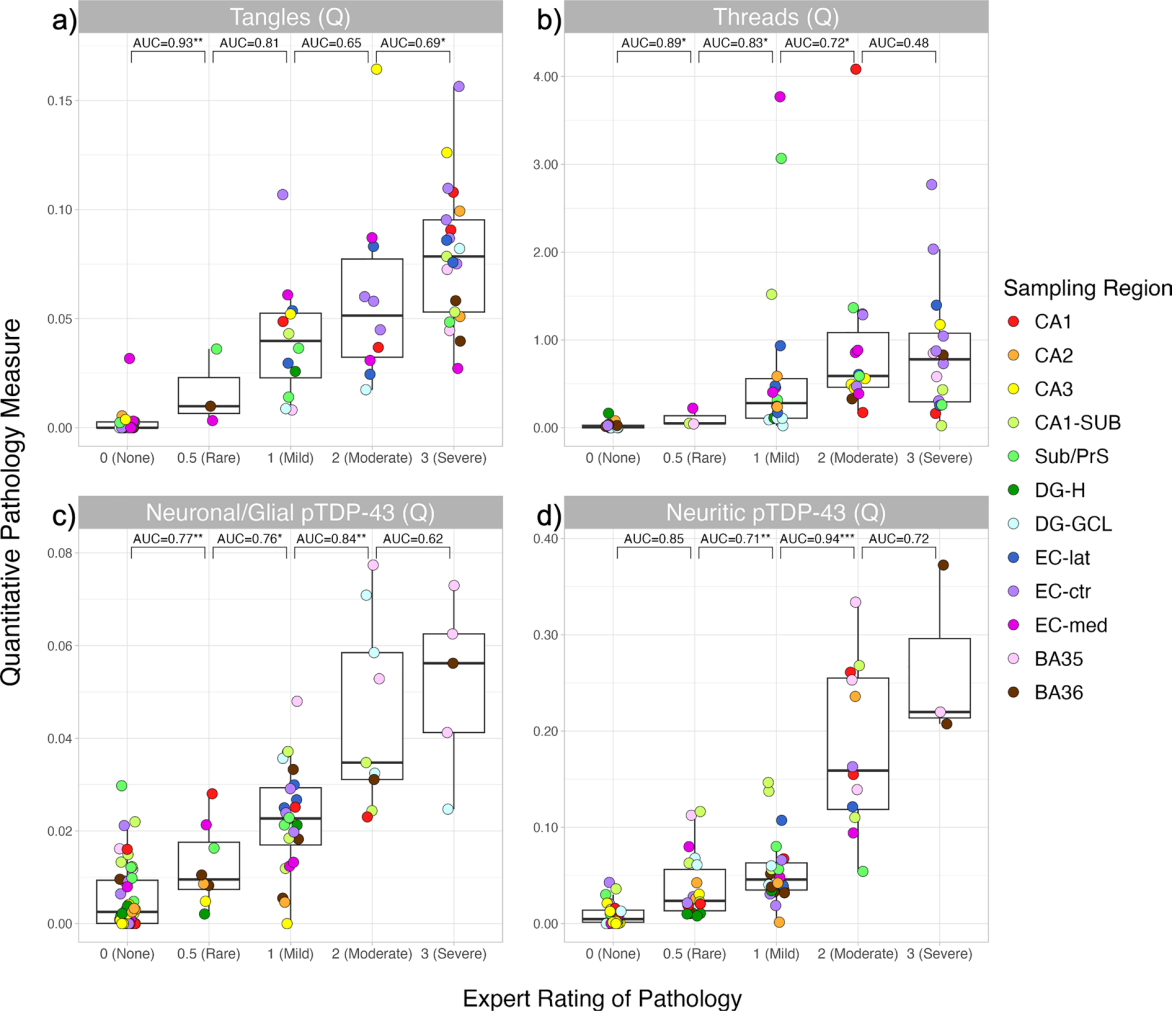
Validation of single sampling region pathology measures with expert ratings. Quantitative pathology measures in individual sampling regions were grouped by the ordinal semi-quantitative rating assigned by a visual read of the same sampling region by an expert (S.A. for p-tau, J.R. for pTDP-43). Each panel represents a different pathology type: tangles (**a**), threads (**b**), neuronal/glial pTDP-43 (**c**), and neuritic pTDP-43 (**d**). The same set of sampling regions were evaluated for tangles and threads, and similarly for the pTDP-43 measures. One-sided AUC and Mann–Whitney U-tests are shown between adjacent ratings (*p < 0.05, **p < 0.01, ***p < 0.001). *CA* cornu ammonis, *CA1-SUB* cornu ammonis 1/subiculum, *SUB/PrS* subiculum/presubiculum, *DG-H* hilus of the dentate gyrus, *DG-GCL* granular cell layer of the dentate gyrus, *EC-lat* lateral portion of entorhinal cortex visible on the section, *EC-ctr* central portion of entorhinal cortex visible on the section, *EC-med* medial portion of the entorhinal cortex visible on the section, *BA* Brodmann area

We tested multiple different summary statistics to generate quantitative pathology measures in individual sampling boxes, including various percentiles, median, and maximum, by comparing these statistics to semi-quantitative ratings of the same boxes by an expert rater. Different measures showed varying discrimination across pathological inclusion types. The 99th percentile was ultimately chosen for all further analyses as it showed high discrimination across all inclusion types (Supplementary Table 3 and Supplementary Figs. 1 and 2). Validation of the quantitative measures using the 99th percentile in individual sampling regions is shown in Fig. 3. The quantitative measures generally showed high discrimination between adjacent categories of semi-quantitative ratings and increased as semi-quantitative ratings increased, but quantitative measures from adjacent semi-quantitative ratings commonly overlapped. Poor separation was seen between the moderate and severe categories for threads (AUC = 0.48). This validation also revealed that *WildCat* incorrectly classified mossy fibers within the dentate gyrus as threads. Therefore, the threads measurements from the hilus of the dentate gyrus were excluded from all summary measures.

### Pathology analyses

Quantitative pathology burden measures in each region of interest were plotted across Braak (Supplementary Fig. 3) and LATE-NC stages (Supplementary Fig. 4). Tangle and thread pathology generally increased in each region across Braak stages; this pattern was particularly clear for tangles. The pattern for pTDP-43 pathology across Braak stages was less consistent and varied by region and inclusion type. The high levels of pTDP-43 pathology, particularly neuritic, in Braak stage 0 patients is likely due to the small sample size (*N* = 4) and driven by one case with stage 2 LATE-NC. Braak stage V/VI cases tended to show higher levels of pTDP-43 pathology than other stages. When examining the LATE-NC staging, p-tau measures often showed slight increases across stages except for LATE-NC stage 3 cases (though note the small sample size for stage 3, *N* = 4). pTDP-43 measures generally showed similarly low levels of pathology for LATE-NC stages 0 and 1 and increasingly higher pathology burden in stages 2 and 3.

**Fig. 4:**
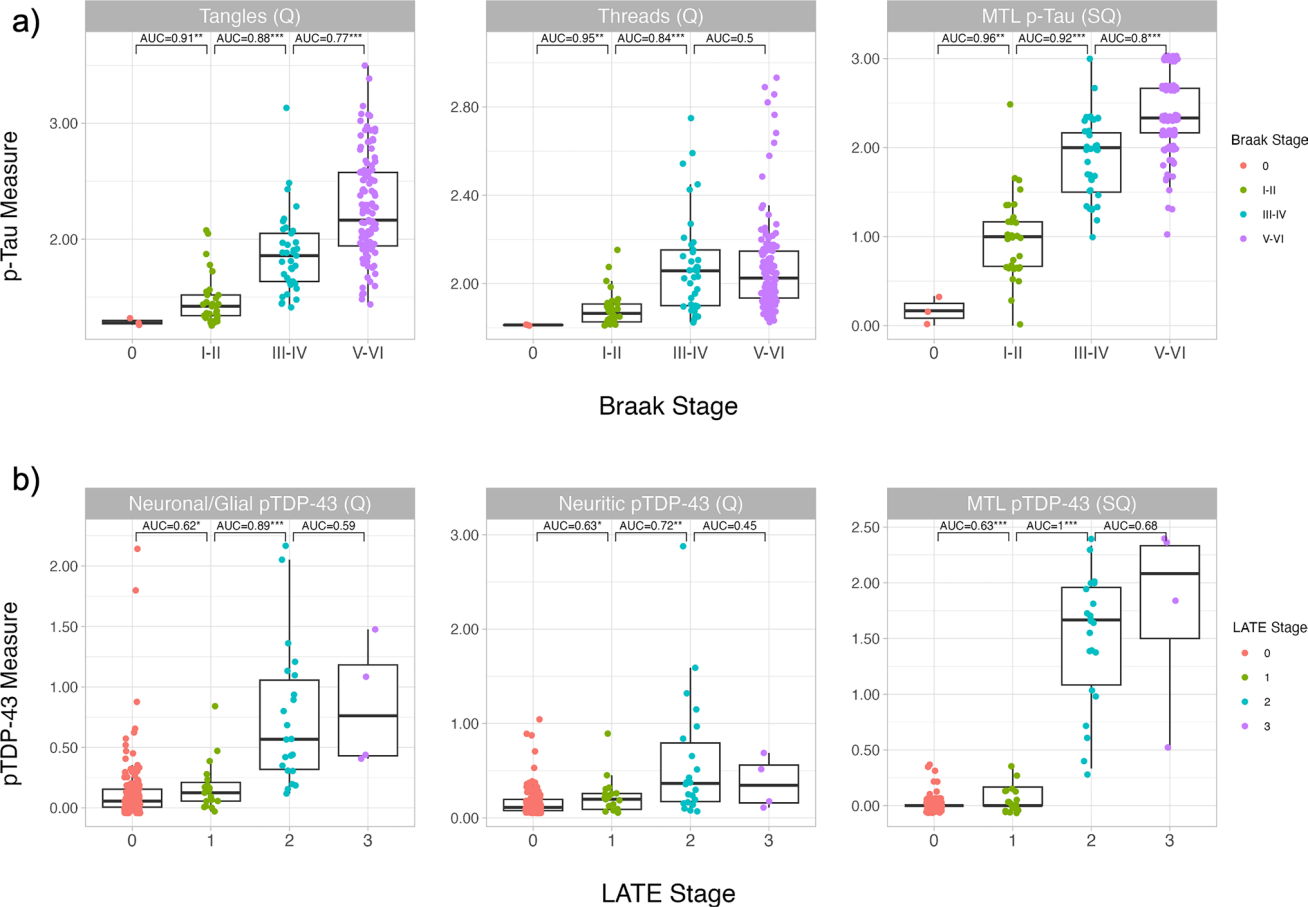
Pathology measures compared to staging systems. **a** Quantitative summary measures and semi-quantitative ratings of p-tau pathology plotted across Braak stages for all patients with quantitative tau summary measurements (N = 196). **b** Quantitative summary measures and semi-quantitative ratings of pTDP-43 pathology plotted across LATE-NC stages for all cases with quantitative pTDP-43 summary measurements (*N* = 190). One-sided AUC and Mann–Whitney *U*-tests are shown between adjacent Braak and LATE-NC stages for each plot (*p < 0.05, **p < 0.01, ***p < 0.001). *Tangles (Q)* quantitative tangles summary measurement, *Threads (Q)* quantitative threads summary measurement, *MTL p-Tau (SQ)* semi-quantitative MTL p-tau rating, *Neuronal/Glial pTDP-43 (Q)* quantitative neuronal/glial pTDP-43 summary measurement, *Neuritic pTDP-43 (Q)* quantitative neuritic pTDP-43 summary measurement, *MTL pTDP-43 (SQ)* semi-quantitative MTL pTDP-43 rating

Both the quantitative pathology summary measures and the semi-quantitative ratings steadily increased across Braak (p-tau) and LATE-NC (pTDP-43) stages and showed varying degrees of separation between stages (Fig. 4). High variation was present in all measures within each Braak stage other than stage 0. The best discriminator between LATE-NC stages was the semi-quantitative MTL pTDP-43 ratings, as expected, as the individual semi-quantitative ratings in this average are also used to define LATE-NC stages. High variation was present within all quantitative summary pTDP-43 measures, including at low stages of LATE-NC. pTDP-43 measures showed low discrimination between stages 0 and 1 and between stages 2 and 3, but higher discrimination between stages 1 and 2.

**Fig. 5:**
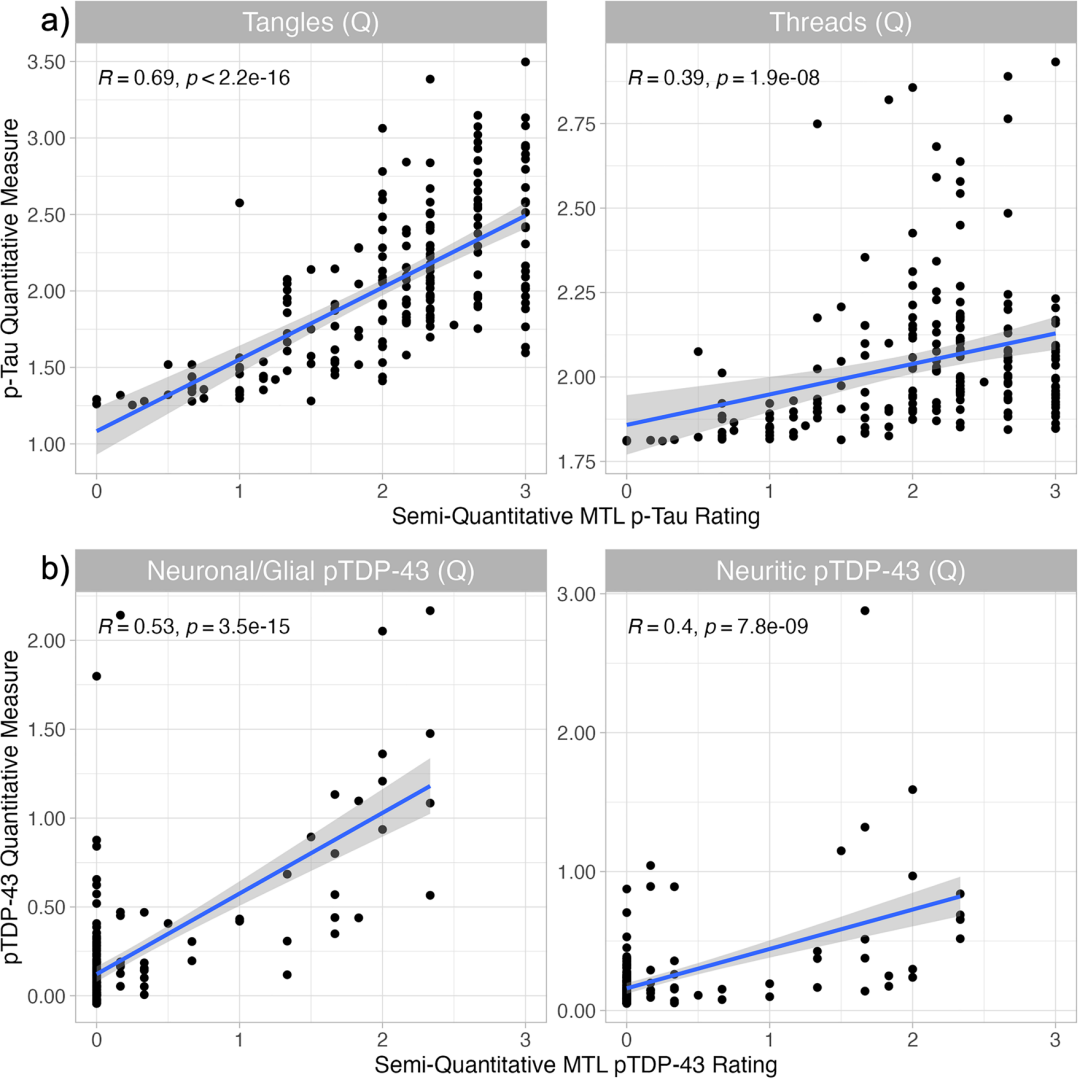
Scatterplots of summary pathology measures and semi-quantitative ratings of the same pathology in the MTL. **a** Scatterplots of the quantitative tangle and threads summary measures plotted against the semi-quantitative MTL p-tau rating (*N* = 196). **b** Scatterplots of the quantitative neuronal/glial and neuritic pTDP-43 summary measures plotted against the semi-quantitative MTL pTDP-43 rating (*N* = 190). Spearman correlations between each summary measure and its corresponding semi-quantitative rating are shown in the top left of each plot. *Tangles (Q)* quantitative tangles summary measurement, *Threads (Q)* quantitative threads summary measurement, *Neuronal/Glial pTDP-43 (Q)* quantitative neuronal/glial pTDP-43 summary measurement, *Neuritic pTDP-43 (Q)* quantitative neuritic pTDP-43 summary measurement

Quantitative pathology summary measures showed varying strength but significant correlations with semi-quantitative ratings of the same MTL pathology; neuronal measures (tangles: *R* = 0.69, *p* < 2.2 × 10^–16^, neuronal/glial pTDP-43: *R *= 0.53, *p* = 3.5 × 10^–15^) showed stronger correlations than neuritic measures (threads: *R* = 0.39, *p* = 1.9 × 10^–8^, neuritic pTDP-43: *R* = 0.40, *p* = 7.8 × 10^–9^) (Fig. 5). Correlations with the average neuronal loss in the MTL followed a similar pattern, though weaker, and the threads summary measure did not significantly correlate with neuronal loss (Supplemental Fig. 5).

### Pathology and imaging

**Fig. 6:**
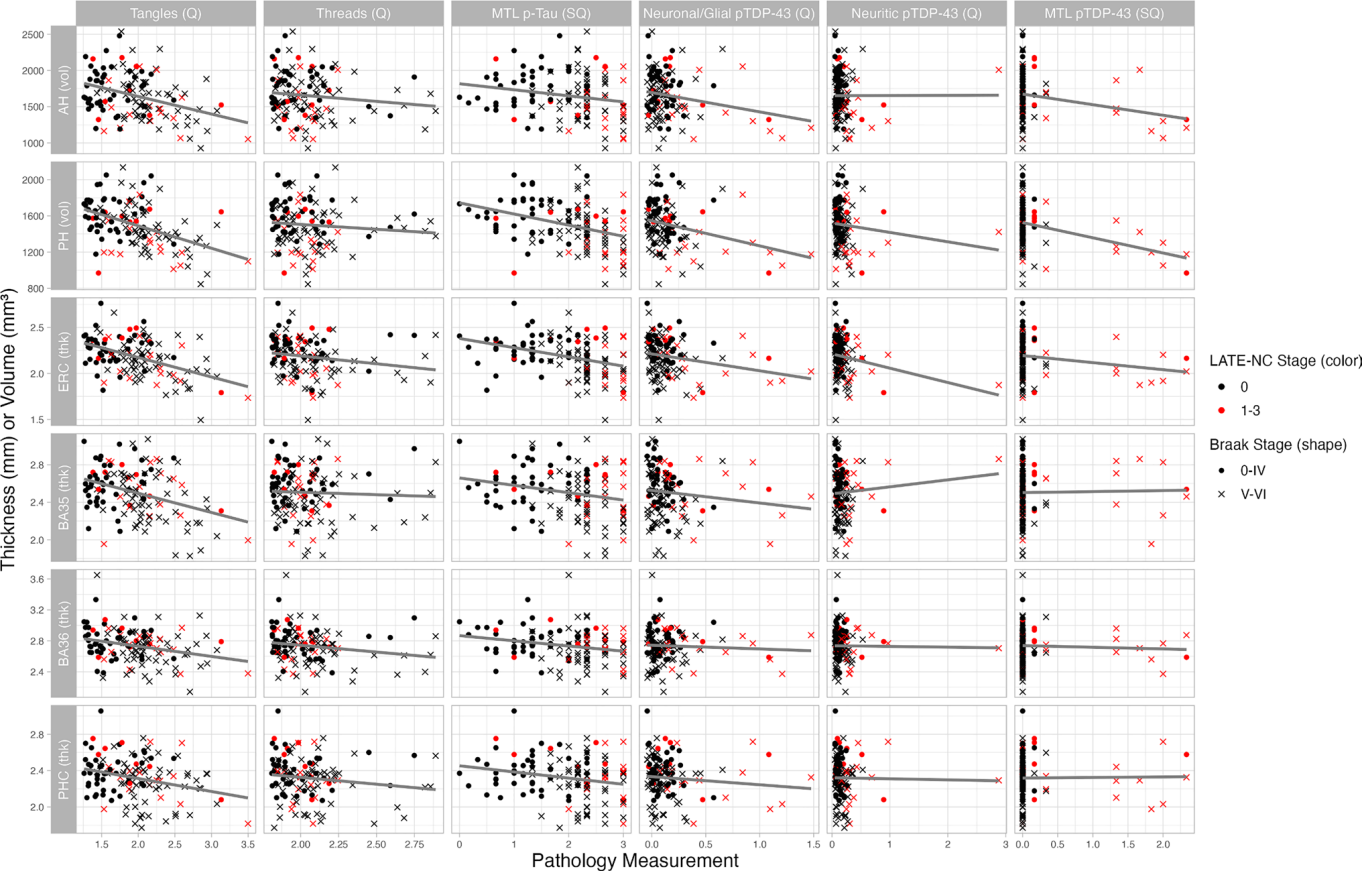
Scatterplots of tau and TDP-43 pathology measures compared to ipsilateral cortical thickness and volume measurements. The sample includes all cases with both quantitative p-tau and pTDP-43 summary pathology measures available and ipsilateral cortical thickness and volume data (*N* = 126). Cases with LATE-NC stages 1–3 (*N* = 24) are colored in red. Cases with Braak stages of V/VI (*N* = 75) are represented by a cross. A regression line without any covariates is also included in each plot. *AH (vol)* anterior hippocampus volume (mm^3^), *PH (vol)* posterior hippocampus volume (mm^3^), *ERC (thk)* entorhinal cortex median thickness (mm), *BA35 (thk)* Brodmann area 35 median thickness (mm), *BA36 (thk)* Brodmann area 36 median thickness (mm), *PHC (thk)* parahippocampal cortex median thickness (mm), *Tangles (Q)* quantitative tangles summary measurement, *Threads (Q)* quantitative threads summary measurement, *MTL p-Tau (SQ)* semi-quantitative MTL p-tau rating, *Neuronal/Glial pTDP-43 (Q)* quantitative neuronal/glial pTDP-43 summary measurement, *Neuritic pTDP-43 (Q)* quantitative neuritic pTDP-43 summary measurement, *MTL pTDP-43 (SQ)* semi-quantitative MTL pTDP-43 rating

Scatterplots between the six MRI ROI-level volume/thickness measures and available ipsilateral quantitative and semi-quantitative pathology measures are plotted in Fig. 6 for the 126 patients for whom all these measures are available (Supplemental Table 4 for demographics). A strong relationship between most MRI measures and the quantitative p-tau tangles summary measure was observed, likely largely driven by individuals with advanced AD (Braak stage V/VI, *N* = 75, labeled with a cross). These individuals exhibited a greater range of variation in the quantitative tangles measure than in the semi-quantitative p-tau measure. All pTDP-43 measures are highly skewed towards zero, reflecting the lack of hippocampal pTDP-43 pathology in most participants. A relationship was also observed between most MRI measures and both the semi-quantitative pTDP-43 rating and the neuronal/glial quantitative pTDP-43 summary measure in the participants with LATE-NC stages 1–3 (*n* = 24, red color).

### Model comparisons

We statistically compared models that included quantitative vs. semi-quantitative measures of p-tau and pTDP-43 burden (Table 3). For each MTL subregion, Table 3 lists the differences in *R*_adj_^2^, prediction error (L1 norm of the residuals), AICc, and BIC between the two non-nested models fitted using the same dependent variable (MRI measures) and nuisance covariates and differing by the type of pathology measures used as the independent variable (quantitative or semi-quantitative). For the p-tau model comparison, we used all cases that had antemortem imaging of the ipsilateral hemisphere and quantitative hippocampal p-tau summary pathology measurements (*N* = 140, Supplemental Table 4 for demographics). The model with quantitative p-tau summary measures had a higher *R*_adj_^2^ and lower prediction error, AICc, and BIC value in every region than the model with semi-quantitative p-tau ratings, except for BIC in the association with PHC. We had positive evidence (∆BIC > 2) that the quantitative model fit better for BA35 and very strong evidence (∆BIC > 10) for AH, PH, and ERC.

**Table 3:** Comparisons of quantitative and semi-quantitative models for p-tau and pTDP-43

p-Tau				
	∆* R*_adj_^2^	∆Prediction Error	∆AICc	∆BIC
AH (volume)	0.084	− 2035.718	− 15.654	− 12.998
PH (volume)	0.111	− 2356.001	− 25.381	− 22.724
ERC (thickness)	0.110	− 1.529	− 18.455	− 15.764
BA35 (thickness)	0.043	− 0.806	− 5.219	− 2.528
BA36 (thickness)	0.027	− 0.572	− 2.829	− 0.138
PHC (thickness)	0.017	− 0.306	− 1.414	1.277

pTDP-43				
	*R*_adj_^2^	∆Prediction error	∆AICc	∆BIC
AH (volume)	− 0.004	− 280.254	1.966	4.641
PH (volume)	− 0.045	437.871	10.568	13.243
ERC (thickness)	0.014	− 0.142	− 1.123	1.586
BA35 (thickness)	0.032	− 0.582	− 3.777	− 1.068
BA36 (thickness)	− 0.004	− 0.003	1.809	4.518
PHC (thickness)	0.013	− 0.429	− 0.944	1.765

For each ROI and pathology, the differences between the models using quantitative or semi-quantitative pathology in adjusted *R*^2^, in prediction error (calculated as L1 norm of the residuals), in AICc, and in BIC were calculated. Note that the large difference in range in prediction error between the hippocampal measures and extracortical regions is likely a result of using volume for AH/PH and thickness for the extracortical regions (ERC, BA35, BA36, PHC). A positive difference in *R*_adj_^2^ favors the quantitative model. A negative difference in prediction error, AICc, and BIC favors the quantitative model. *AH* anterior hippocampus volume, *PH* posterior hippocampus volume, *ERC* entorhinal cortex median thickness, *BA35* Brodmann area 35 median thickness, *BA36* Brodmann area 36 median thickness, *PHC* parahippocampal cortex median thickness. *AICc* Akaike information criterion with correction for small sample sizes, *BIC* Bayesian information criterion

The model comparisons for quantitative vs. semi-quantitative pTDP-43 (*N* = 142, Supplemental Table 4 for demographics) showed much less consistency across MTL regions. Quantitative models produced a higher *R*_adj_^2^ for three of the four cortical regions, but not either hippocampal region, and had a smaller prediction error for every region other than PH. AICc values favored quantitative pathology measures for ERC, BA35, and PHC, but these effects were weak or in the opposite direction when using BIC values. BIC values showed positive evidence (∆BIC > 2) favoring the semi-quantitative measures for BA36 and AH, and very strong evidence (∆BIC > 10) favoring the semi-quantitative model for PH.

Overall, the model comparison suggests that quantitative hippocampal p-tau summary measures provide a better fit to the MTL structural model than the semi-quantitative p-tau rating. However, for pTDP-43 there is no such clear advantage of using quantitative measures, and indeed, semi-quantitative rating may be preferable. Based on this conclusion, we include quantitative p-tau summary measurements and semi-quantitative MTL pTDP-43 ratings in the thickness-to-structure analyses in the following sections.

### Pathology and structure analyses

140 patients had ipsilateral imaging and quantitative p-tau hippocampal summary pathology measurements available and were included in these analyses (Supplemental Table 4 for demographics). T-statistics from the linear models fitted in this entire cohort and in the advanced ADNC and LATE-NC subgroups are shown in Table 4. In the whole cohort, the tangles summary measures showed significant negative associations with all regions, with the strongest effects in absolute terms with ERC (*β* =  − 0.47). Significant associations were also found between the MTL pTDP-43 rating and smaller volumes and thickness in AH, PH, ERC, and BA36, but the β coefficients were smaller in absolute terms than the associations with the tangles measure. The strongest association of pTDP-43 was with PH (*β* =  − 0.30). In the LATE-NC subgroup (*N* = 31), the tangles summary measure showed significant negative associations with AH, PH, and ERC, and this association was strongest with ERC (*β* =  − 0.67). Additionally, significant associations were found between the MTL pTDP-43 rating and all regions except BA35 and PHC, and the strongest effect was with PH (*β* =  − 0.48), though AH and ERC also showed similarly high associations (*β* =  − 0.47 for both). We repeated these analyses in the LATE-NC stage 1 and 2/3 patients separately (Supplementary Table 5). Here, we found multiple significant associations with p-tau (AH, ERC, and BA35) but not with pTDP-43 in the LATE-NC stage 1 patients (*N* = 17) and no significant associations with either pathology in the LATE-NC stage 2/3 patients (*N* = 14). Finally, in the advanced ADNC subgroup (*N* = 85), the quantitative tangles summary measure was again significantly associated with smaller volumes and thickness in all subregions except PHC and BA36, with the strongest association with PH (*β* = − 0.51). Finally, the MTL pTDP-43 rating also showed significant associations with AH, PH, and ERC, which was strongest for PH (*β* = − 0.26).

**Table 4:** Pathology to structure analyses in cases with quantitative p-tau pathology measures and ipsilateral MRI

	AH (vol)	PH (vol)	ERC (thk)	BA35 (thk)	BA36 (thk)	PHC (thk)
Whole cohort (*N* = 140, df = 131/132)						
Tangles (Q)	***β* = − 0.30**,	***β* = − 0.44,**	***β* = − 0.47,**	***β* = − 0.31,**	***β* = − 0.23,**	***β* = − 0.27,**
	***t* = − 3.94**,	***t* = − 6.37,**	***t* = − 5.84,**	***t* = − 3.41,**	***t* = − 2.51,**	***t* = − 3.01,**
	***p* = 6.50e-05**	***p* = 1.48e-09**	***p* = 1.97e-08**	***p* = 4.28e-04**	***p* = 0.007**	***p* = 0.002**
Threads (Q)	*β* = − 0.07,	*β* = − 0.01,	*β* = 0.01,	*β* = 0.09,	*β* = − 0.10,	*β* = − 0.01,
	*t* = − 0.88,	*t* = − 0.16,	*t* = 0.17,	*t* = 0.99,	*t* = − 1.14,	*t* = − 0.07,
	*p* = 0.190	*p* = 0.436	*p* = 0.566	*p* = 0.838	*p* = 0.129	*p* = 0.473
MTL pTDP-43 (SQ)	***β* = − 0.28,**	***β* = − 0.30,**	***β* = − 0.26,**	*β* = − 0.04,	*β* = *− 0.16,*	*β* = − 0.08,
	***t* = − 3.91,**	***t* = − 4.73,**	***t* = − 3.46,**	*t* = − 0.50,	*t* = *− 1.92,*	*t* = − 0.98,
	***p* = 7.29e-05**	***p* = 2.86e-06**	***p* = 3.58e-04**	*p* = 0.308	*p* = *0.028*	*p* = 0.164
LATE-NC Cohort (*N* = 31, df = 22/23)						
Tangles (Q)	***β* = − 0.47,**	*β* = *− 0.37,*	***β* = − 0.67,**	* β* = − 0.35,	* β* = − 0.14,	* β* = − 0.26,
	***t* = − 3.01,**	*t* = *− 1.82,*	***t* = − 3.79,**	*t* = − 1.38,	*t* = − 0.59,	*t* = − 1.22,
	***p* = 0.003**	*p* = *0.041*	***p* = 4.72e-04**	*p* = 0.091	*p* = 0.281	*p* = 0.118
Threads (Q)	*β* = 0.33,	*β* = 0.14,	*β* = 0.26,	*β* = 0.21,	*β* = 0.07,	*β* = 0.08,
	*t* = 2.42,	*t* = 0.79,	*t* = 1.64,	*t* = 0.92,	*t* = 0.33,	*t* = 0.44,
	*p* = 0.988	*p* = 0.782	*p* = 0.942	*p* = 0.818	*p* = 0.629	*p* = 0.668
MTL pTDP-43 (SQ)	***β* = − 0.47,**	***β* = − 0.48,**	***β* = − 0.47,**	* β* = − 0.12,	* β* = *− 0.37,*	* β* = − 0.18,
	***t* = − 3.58,**	***t* = − 2.76,**	***t* = − 3.16,**	*t* = − 0.55,	*t* = *− 1.77,*	*t* = − 1.02,
	***p* = 8.41e-04**	***p* = 0.006**	***p* = 0.002**	*p* = 0.293	*p* = *0.045*	*p* = 0.160
Advanced ADNC cohort (N = 85, df = 76/77)						
Tangles (Q)	***β* = − 0.37,**	***β*** **= − 0.51,**	***β* = − 0.45,**	* β* = *− 0.24,*	* β* = − 0.18,	* β* = − 0.17,
	***t* = − 3.88,**	***t*** = **− 6.27**,	***t* = − 4.37,**	*t* = *− 2.11,*	*t* = − 1.63,	*t* = − 1.44,
	***p* = 1.09e-04**	***p*** = **9.85e-09**	***p* = 1.89e-05**	*p* = *0.019*	*p* = 0.054	*p* = 0.078
Threads (Q)	*β* = − 0.13,	*β* = − 0.02,	*β* = − 0.06,	*β* = 0.03,	*β* = − 0.18,	*β* = − 0.02,
	*t* = − 1.38,	*t* = − 0.26,	*t* = − 0.61,	*t* = 0.26,	*t* = − 1.63,	*t* = − 0.15,
	*p* = 0.085	*p* = 0.399	*p* = 0.270	*p* = 0.602	*p* = 0.054	*p* = 0.442
MTL pTDP-43 (SQ)	*β* = *− 0.21,*	***β* = − 0.26,**	*β* = *− 0.22,*	*β* = 0.08,	*β* = − 0.04,	*β* = − 0.02,
	*t* = *− 2.22,*	***t* = − 3.07,**	*t* = *− 2.11,*	*t* = 0.71,	*t* = − 0.32,	*t* = − 0.15,
	*p* = *0.015*	***p* = 0.001**	*p* = *0.019*	*p* = 0.759	*p* = 0.375	*p* = 0.440

The standardized β coefficient, t statistic, and p value for the association of each pathology measure with each imaging ROI is given within each cohort (top: whole cohort, *N* = 140, middle: subset of cases with LATE-NC stages 1–3, *N* = 31, bottom: subset of cases with Braak Stage V/VI, *N* = 85). Degrees of freedom (df) are also given for each cohort; the first number is for AH/PH associations due to the additional covariate of ICV, while the second number is for ERC, BA35, BA36, and PHC associations. Associations that would survive Bonferroni correction (*P* < 8.33e-03) are bolded; associations that are significant at *P* < 0.05 but would not survive corrections are italicized. *AH (vol)* anterior hippocampus volume, *PH (vol)* posterior hippocampus volume, *ERC (thk)* entorhinal cortex median thickness, *BA35 (thk)* Brodmann area 35 median thickness, *BA36 (thk)* Brodmann area 36 median thickness, *PHC (thk)* parahippocampal cortex median thickness, *Tangles (Q)* quantitative tangles summary measure, *Threads (Q)* quantitative threads summary measure, *MTL pTDP-43 (SQ)* semi-quantitative MTL pTDP43 rating

While model comparisons showed quantitative p-tau and semi-quantitative pTDP-43 measures were the better models of thickness, for completeness, in Supplemental Tables 6, 7, and 8, we also show these pathology-structure associations in each group including the semi-quantitative MTL p-tau rating or quantitative pTDP-43 summary measures. Notably, in the Braak V/VI patients, weak associations with the semi-quantitative p-tau rating were only statistically significant in two regions, compared to the quantitative p-tau tangles summary measure which showed strong associations across the MTL. In Supplemental Table 9, we also repeated the models in cases with an antemortem interval of less than 3 years and found similar results of widespread, significant associations of tau tangles compared to more limited associations of MTL subregions with TDP-43.

### Pointwise regional thickness associations

**Fig. 7:**
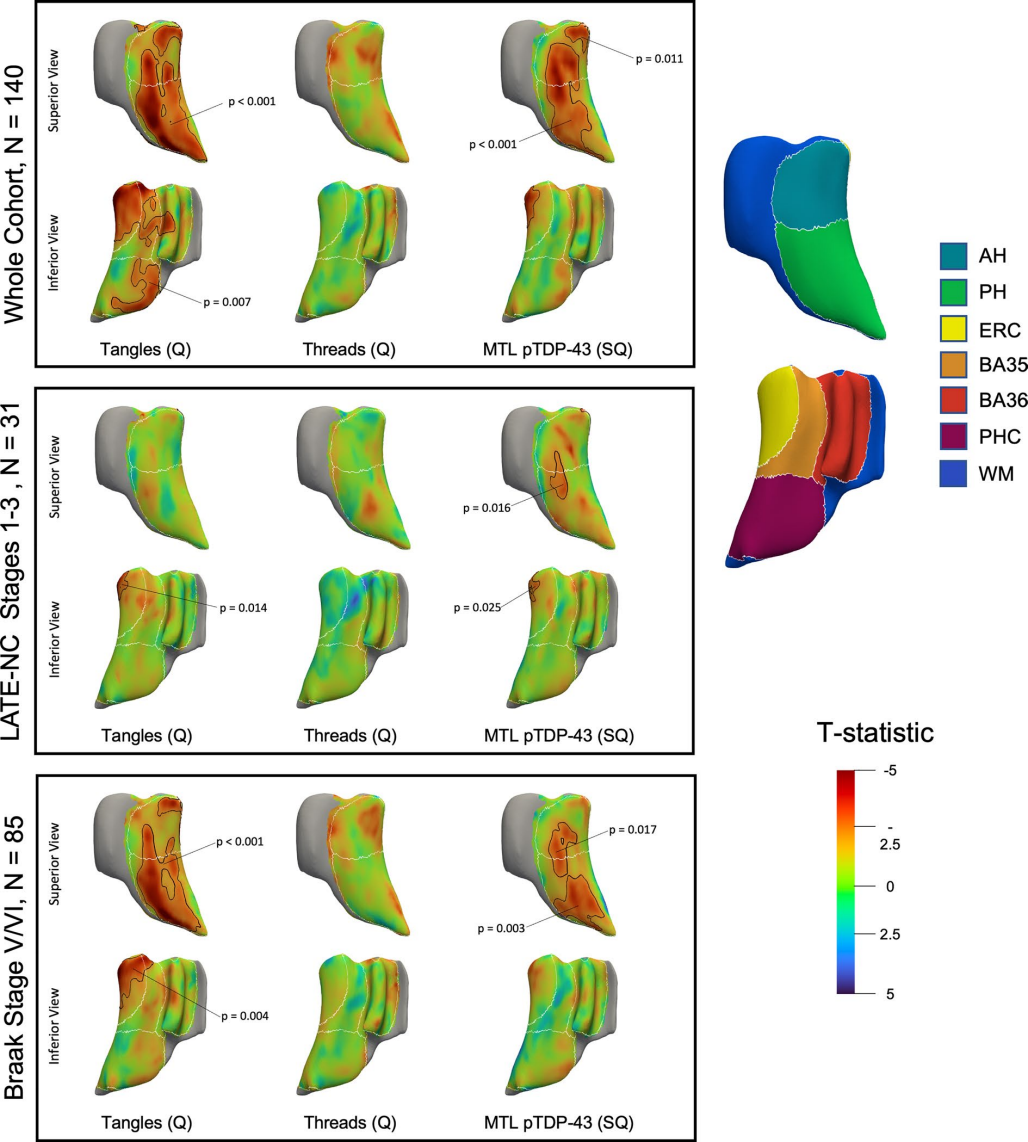
Maps showing the association between pointwise cortical thickness and the tangles summary measure, threads summary measure, and the MTL pTDP-43 semi-quantitative rating. Each row represents a different cohort; top: the whole cohort (*N* = 140), middle: the subset of patients with LATE-NC stages 1–3 (*N* = 31), and bottom: the subset of patients with Braak staging of V/VI (*N* = 85). Each model is covaried for age, antemortem interval, MRI field strength, and sex. The clusters outlined in black indicate regions where a significant association was found (*p* < 0.05), and the *p*-values are indicated with a line. The white lines represent the borders between subfields. To the right, a 3-D representation of the MTL with the subregions labeled is shown; the white matter sections were not analyzed. Warm colors indicate areas with more negative t-statistics. *AH* anterior hippocampus, *PH* posterior hippocampus, *ERC* entorhinal cortex, *BA35* Brodmann area 35, *BA36* Brodmann area 36, *PHC* parahippocampal cortex, *WM* white matter

Pointwise regional thickness association maps showed similar patterns to the ROI-level linear models (Fig. 7). In the whole cohort, the tangles summary measure showed strong associations across almost the entirety of the hippocampus and much of the extrahippocampal regions. We also found strong associations between the pTDP-43 rating and thickness across the hippocampus and in the entorhinal cortex. In the LATE-NC stages 1–3 subgroup, the tangles summary measure was significantly associated with thickness in a small portion of the entorhinal cortex. The pTDP-43 rating showed significant associations in the hippocampus and entorhinal cortex. Finally, in the advanced ADNC subgroup, the tangles summary measure again showed significant associations in the hippocampus, entorhinal cortex, and BA35, and the pTDP-43 measure showed associations only in the hippocampus. Similar to the ROI-level analyses, we also computed these pointwise analyses in additional models including the quantitative pTDP-43 or semi-quantitative p-tau measures (Supplementary Fig. 6) and found similar results.

## Discussion

In the current study, we developed and validated algorithms to quantify postmortem p-tau and pTDP-43 pathology and evaluated the associations of these pathologies with MTL neurodegeneration. We show that quantitative pathology measurements show high accuracy across multiple validation experiments and discriminate well between stages of pathology. We found that quantitative p-tau and semi-quantitative pTDP-43 measures were the best performing models of antemortem structural measures. In 140 cases with antemortem neuroimaging, we investigated the association of these pathology measures with MTL subregional thickness and volume measures. In both the ROI-level linear models and pointwise regional thickness analyses, we find significant associations between the hippocampal p-tau tangles summary measure and all MTL subregions, with the strongest associations in absolute terms with ERC, PH, and AH. Associations with pTDP-43 pathology were generally limited to AH, PH, and ERC.

Our findings of widespread p-tau associations are consistent with prior studies using both antemortem and postmortem imaging and pathology measures [3, 20, 77, 96, 98]. Model comparisons in the whole cohort showed very strong evidence that the quantitative p-tau summary pathology measures were a better model of antemortem structural measures than the semi-quantitative p-tau ratings for AH, PH, and ERC, and showed at least weak evidence in favor of quantitative measures for all MTL subregions. Additionally, within cases with Braak stages of V/VI, the tangles summary measurement was significantly associated with subregions across the MTL, while the semi-quantitative MTL p-tau rating showed weakened relationships that were only significantly associated with smaller PH volume and ERC thickness. These distinctions are consistent with our hypothesis that quantitative pathology measurements will reduce the ceiling effect observed in semi-quantitative ordinal ratings and provide more granular information about local pathological burden. In the advanced ADNC subgroup, most of the semi-quantitative ratings were very high, while the quantitative measurements showed a large variance in pathological burden, which, according to our results, enhances the ability to predict structural changes beyond coarser stage-based or semi-quantitative measurements. This effect, however, was only seen with tangles; the threads summary measure showed only a few weak but significant associations with antemortem structure. Validation with expert ratings showed poor separation between moderate and severe ratings of thread pathology, suggesting that the threads measurement may be overwhelmed with high amounts of pathology. Of the four pathologies measured quantitatively, the threads measure also showed the lowest accuracy. We also note that, had we used two-tailed tests or a one-sided test for positive associations, a positive association would have been found between the threads measure and AH in the LATE stages 1–3 subgroup. This finding is likely spurious given the small sample size and comparatively weak β coefficients. Future work is needed to understand whether these weak effects are due to limitations of the threads measurement itself or to underlying biology.

The results using quantitative pathology methods for pTDP-43 were less conclusive. Consistent with prior literature [2, 10, 45, 47, 96] we found the strongest associations of pTDP-43 pathology (measured both quantitatively and semi-quantitatively) with AH, PH, and ERC in both our whole cohort and analyses restricted to LATE patients. This pattern was particularly clear in our pointwise regional thickness association maps, where p-tau associations spanned the MTL, but pTDP-43 associations were limited to only the hippocampus and entorhinal cortex. While quantitative p-tau measurements improved upon semi-quantitative ratings in modeling antemortem structural measures, quantifying pTDP-43 did not produce the same advantage. Model comparisons showed only weak evidence that quantitative TDP-43 summary pathology measurements improved model fit for some extrahippocampal regions, and for some regions semi-quantitative measurements were a significantly better model of thickness. We have several hypotheses for this distinction.

First, prior work has shown that in cases with both ADNC and TDP-43 pathology, the vast majority of immunoreactivity is from tau pathology, with only a small amount due to TDP-43 inclusions [63]. Thus, even in cases with LATE-NC, there are likely much fewer pTDP-43 inclusions in a given section than there are p-tau inclusions in an AD case. As a result, our sampling strategy, which did not sample the entire hippocampus, may have missed some of the pTDP-43 pathology that was captured by the more holistic semi-quantitative ratings. Additionally, unlike p-tau, the semi-quantitative pTDP-43 ratings did not show a ceiling effect; variance in both quantitative and semi-quantitative pTDP-43 measures was high, even at severe LATE-NC stages. As a result, the semi-quantitative pTDP-43 ratings may be sufficient to capture the variance associated with thickness.

We also note that all four quantitative measures were associated with some noise, but this may be a larger issue for pTDP-43 analyses because of the imbalance in our cohort. Specifically, only 29 of the 142 cases examined in the pTDP-43 model comparisons had any stage of LATE-NC, and only 14 had LATE-NC stages 2 or 3, where hippocampal pTDP-43 pathology was expected. The noise in the quantitative pTDP-43 measures in the large number of cases with no/low LATE-NC may have drowned out the real signal in the smaller subset of cases that were expected to have any hippocampal pTDP-43 pathology (LATE-NC stage 2/3). The semi-quantitative ratings, however, had little to no noise at low LATE-NC stages, which could improve the strength of this association.

Finally, our quantitative measurements of pTDP-43 in LATE-NC may have also been confounded by the fact that pTDP-43 is associated with neuronal loss and no remnant of the pTDP-43 inclusions after cell death, as opposed to the presence of ghost tangles with p-tau NFTs. Indeed, in FTLD-TDP-43 type C cases, Kawles et al. [52] found that MTL regions showed the highest levels of neurodegeneration but with low densities of TDP-43 inclusions at autopsy; areas without significant neurodegeneration showed higher densities of TDP-43 inclusions. This association might result in lower amounts of pTDP-43 in cases with more severe neuronal loss which may blunt relationships between structure and total pTDP-43 load. Staging schemes, which are a measure of extent, may be less sensitive to this process.

There are also some statistical reasons for this distinction: the model comparisons were influenced by differences in the number of parameters in the models. The semi-quantitative model only had one pTDP-43 variable (semi-quantitative MTL pTDP-43 rating) while the quantitative model had two (neuronal/glial and neuritic pTDP-43 summary measures), and both BIC and AICc penalize models with more parameters. Including only the neuronal/glial pTDP-43 variable in the quantitative model slightly improved the model relative to the semi-quantitative model, but not substantially.

This study builds on findings from our previous work [27, 95]. We have an overlapping but distinct sample from these studies with newer autopsies included, cases with any neuropathological diagnosis of non-AD tauopathies and FTLD excluded, and only ipsilateral MRI and pathology measures examined. Additionally, we now utilize both semi-quantitative and quantitative measurements of pathology to characterize regional pathology burden. The previous studies did not find as widespread associations with tau as the current study; explanations for this distinction include differences in statistical analysis (such as correction for multiple comparisons and the inclusion of other pathologies), sample differences, and improved ability in the present study to measure p-tau burden via quantitative methods. In addition, de Flores and colleagues [27] analyzed patients with intermediate and high ADNC and Wisse et al. [95] analyzed patients who were beta-amyloid negative, whereas here we examined patients across all levels of ADNC. As a result, we likely had larger variation in the amount of p-tau present; if we limited our sample to only the intermediate and high ADNC cases, like de Flores et al., most of those cases would fall in the Braak V/VI analysis, where we found more limited associations of the semi-quantitative MTL p-tau rating with structure.

Both prior works also showed a clear relationship of TDP-43 pathology to AH atrophy, which has been found in other studies as well [2, 33, 77], and is consistent with descriptions of pathology progression in LATE-NC beginning in the anterior MTL [45, 46, 69]. However, in our study, we observed statistically significant associations of pTDP-43 with AH, PH, and ERC, and there was no clear predilection for preferential association of pTDP-43 with AH. Multiple LATE-NC stage 2/3 cases were excluded from the imaging analyses due to failed segmentation, potentially increasing the likelihood of exclusion of cases with more severe AH atrophy. These exclusions could have contributed to the discrepancies with the previously documented associations of TDP-43 with AH neurodegeneration [2, 27, 33, 77, 95].

There are a few limitations of this study. First, we were unable to analyze associations between structure and pathology at the level of individual ROIs. Due to tearing and staining, not every region was annotated in every histology section, so analyses mapping pathology regions to MRI regions would have substantially reduced our sample size. Instead, we averaged ROI-level pathology measures in the hippocampus for summary quantitative measures. We also pursued slightly different methods of annotations for the two pathologies. For p-tau, novices and experts met and annotated cases in a one-day rating event, and all slides from each specimen were available for annotations. For pTDP-43, novices were trained and supervised by experts and subsequently performed annotations over the course of a few months, and sections specific to different diseases were prioritized for training. [Many of the annotators for the p-tau training event had participated in prior similar training events [103] and were familiar with p-tau pathology. pTDP-43 annotations were new to all other than the experts, so we elected to train a smaller number of researchers over a longer training period to annotate pTDP-43 inclusions]. Differences in *WildCat* performance are thus likely a result of both biological differences and training protocols.

As stated previously, we also excluded some severely atrophied LATE-NC cases due to mis-segmentation by T1-ASHS. The original data-set used to train T1-ASHS was composed of cognitively normal cases and mildly cognitive impaired patients [104]. Thus, ASHS failed to accurately segment some of the more severely atrophied cases with LATE-NC or more severe AD. These segmentation failures reduced the size of the LATE-NC imaging cohort, which could have contributed to the weaker associations seen with pTDP-43, particularly in the LATE-NC stage 2/3 supplementary analysis where the sample size was limited (*N* = 14). The rate of LATE-NC in this study (22% in the main analyses with any stage of LATE-NC) was lower than reported in other studies. For example, in a large multi-cohort study of over 6000 participants, Nelson et al. (2022) found that 39% of cases had LATE-NC [67]. This discrepancy is likely attributed to a lower age in our study (average age of 75 years) compared to prior studies (88 in [67]), as LATE-NC is more prevalent in participants of older age [68, 70]. Additionally, we used ordinal ratings of MTL pathology to define LATE-NC stages, but this was not a perfect concordance with current staging guidelines [69] due to inconsistencies in the location of the entorhinal cortex rating (at the level of the amygdala or hippocampal body) and the fact that our semi-quantitative ratings examine all pTDP-43 pathology, not just NCIs. This creates some ambiguity in staging, mostly between stages 1 and 2, but is unlikely to have had a major influence on our results.

We view the use of antemortem MRI as a strength of this study but recognize that other imaging modalities could also be used with distinct advantages and disadvantages for each. Ex vivo MRI allows for much higher resolution images and captures MRI and pathology measures at the same time point. However, ex vivo MRI is not widely performed, and its analysis must cope with changes to the brain due to swelling, brain removal, and fixation. Studies leveraging antemortem MRI are easier to generalize to in vivo biomarkers, but disadvantages include variability in quality, protocol, and availability of antemortem MRI data, as well as potentially long-time intervals between MRI and pathology measures, unless imaging is performed in a terminally ill population [14, 15]. We limit our scans to only those within 10 years before death and covary for this antemortem interval, but this approach cannot fully account for differences in disease progression between the time of antemortem MRI and histopathology. A compromise approach is to scan cadavers before autopsy using in vivo MRI protocols, but this approach is only possible in very few select medical centers [41, 42]. We chose to use antemortem imaging to maximize our sample size and increase the feasibility of generalizing our findings to in vivo biomarkers.

This study also has some additional strengths. First, we used postmortem neuropathology and antemortem MRI of the ipsilateral hemisphere. Our large cohort is well-characterized in terms of neuropathology; we exclude any cases with neuropathological diagnoses of FTLD, ALS, or non-AD tauopathies, such that our results are specific to p-tau and pTDP-43 in the context of AD and LATE-NC. Critically, we utilize both quantitative and semi-quantitative measurements of neuropathology, which allows us to reduce the ceiling effect in severe tau pathology populations seen with semi-quantitative ratings. We considered associations significant at *p* < 0.05 due to strong a priori hypotheses about the negative relationship between pathology and antemortem structural measures, but the majority of these associations would also survive Bonferroni correction for multiple comparisons (*p* < 8.33e-03, corresponding to 0.05/6 regions, bolded in each table). While reporting uncorrected results can increase the likelihood of type I errors, the fact that most results here would survive correction for multiple comparisons suggests that chance is small. Future work should expand the sample to include more cases with LATE-NC to re-evaluate our pTDP-43 findings, including the model comparisons, in a larger cohort. Additionally, as many of the cases with LATE-NC had pTDP-43 pathology limited to the amygdala, and TDP-43 has been also shown to be associated with amygdala volume [60], future studies should examine the relationship between imaging and pathology measures of the amygdala as well.

In conclusion, we find strong associations between quantitative measurements of hippocampal p-tau tangles with antemortem MRI measures across MTL subfields, compared to a more limited effect of pTDP-43 to the hippocampus and entorhinal cortex. These different patterns of association can be used to advance in vivo imaging markers of tau and TDP-43 pathology. While quantitative and semi-quantitative measures of pTDP-43 performed similarly, there was a clear improvement in modeling thickness using quantitative measurements of p-tau pathology and reduced ceiling effect in severe disease stages. Taken together, the results demonstrate the power of detailed semi-quantitative and quantitative neuropathology and antemortem neuroimaging analyses to identify neurodegeneration patterns of common co-pathologies and motivate continued investigation to improve characterization of these patterns in vivo.

## Supplementary Information

Below is the link to the electronic supplementary material.

Supplementary Information (DOCX 11290 KB)

Imaging Analyses Data (CSV 115 KB)

Pathology Analyses Data (CSV 137 KB)

## Data Availability

Pathology and imaging measurements have been uploaded as supplementary material and to Dryad (https://doi.org/10.5061/dryad.95x69p8tq). Images will be uploaded to the same depository pending anonymization.
