# Supplementary material for: Association of quantitative histopathology measurements with antemortem medial temporal lobe cortical thickness in the Alzheimer’s disease continuum
Source: Acta Neuropathol. 2024 Sep 3;148(1):37. doi: 10.1007/s00401-024-02789-9 (PMC11371872; doi:10.1007/s00401-024-02789-9)
Supplement: Supplementary file 1 — Supplementary Information (DOCX 11290 KB) [file 401_2024_2789_MOESM1_ESM.docx]

Association of quantitative histopathology measurements with antemortem medial temporal lobe cortical thickness in the Alzheimer’s Disease continuum

For submission to Acta Neuropathologica

Amanda E. Denning, Ranjit Ittyerah, Lisa M. Levorse, Niyousha Sadeghpour, Chinmayee Athlaye, Eunice Chung, Sadhana Ravikumar, Mengjin Dong, Michael Tran Duong, Yue Li, Ademola Ilesanmi, Lasya P. Sreepada, Philip Sabatini, MaKayla Lowe, Alejandra Bahena, Jamila Zablah, Barbara E. Spencer, Ryohei Watanabe, Boram Kim, Maja Højvang Sørensen, Pulkit Khandelwal, Christopher Brown, Stanislau Hrybouski, Sharon X. Xie, Robin de Flores, John L. Robinson, Theresa Schuck, Daniel T. Ohm, Sanaz Arezoumandan, Sílvia Porta, John A. Detre, Ricardo Insausti, Laura E.M. Wisse, Sandhitsu R. Das, David J. Irwin, Edward B. Lee, David A. Wolk, Paul A. Yushkevich.

Correspondence to: Amanda E. Denning, Department of Radiology, University of Pennsylvania, Suite D601, Richards Building, 3700 Hamilton Walk, Philadelphia, PA 19104, USA.

Email: amanda.denning@pennmedicine.upenn.edu

**1. Supplemental Methods**

**Tau Pathology Training Data**

Slides from 37 specimens across neuropathological diagnoses (including Alzheimer’s disease (AD), non-AD tauopathies, frontotemporal lobar degeneration with TDP-43 inclusions (FTLD-TDP-43), limbic predominant age-related TDP-43 encephalopathy (LATE), and Lewy Body Disease (LBD)) and Thal, Braak, and CERAD stages were selected for annotation. Cases with primary neuropathological diagnoses of a non-AD tauopathy (i.e., Pick’s disease, progressive supranuclear palsy, corticobasal degeneration) with a CERAD score greater than 0 were excluded due to the difficulty of distinguishing astrocytic p-tau from neuritic plaques in these cases. All slides for each selected specimen were available to annotate; raters were given randomly assigned lists of slides. Regularly sampled regions at the Penn Center for Neurodegenerative Disease Research (CNDR) include middle frontal cortex, superior temporal cortex, angular gyrus, occipital cortex, anterior cingulate, motor cortex, amygdala, hippocampus, thalamus, substantia nigra, pons, medulla, and dentate nucleus. The spinal cord was also sampled at levels of the cervical, thoracic, lumbar, and sacral spinal cord in some cases. 118 PHF1-stained sections were annotated in total.

**p-Tau Pathology Annotation Criteria**

The following criteria were developed as descriptions of each type of inclusion. Criteria was developed with expert pathologists before the training event and then reviewed and edited the day of annotations. The bolded text indicates the name of the label. Note that the categories here do not correspond to the classes used in final training.

- Neuronal soma / perikaryon inclusions:
  - **Gray matter neurofibrillary tangle:** pyramidal-shaped neurofibrillary tangle
  - **Gray matter non-pyramidal tangle:** non-pyramidal neuronal tangle, more circular than a neurofibrillary tangle
  - **Gray matter pretangle:** pretangle, granular and irregularly dense neuronal inclusion, faint with more variation in intensity
  - **Gray matter ballooned neuron:** swollen, often pyramidal-shaped neuronal inclusion in deep layers closer to white matter, fainter staining in middle is possible
- Dendrites / axons:
  - **Gray matter threads:** area of p-tau threads in gray matter
  - **Gray matter neuritic plaque:** neuritic plaque, clusters of round and sausage-shaped inclusions, larger than a single tangle
  - **Gray matter tangle associated neuritic cluster:** tangle associated neuritic cluster; threads and dot-like inclusions but not in the shape of a tangle, more common in hippocampus but rare in general
  - **Gray matter grains:** gray matter punctuate, dot-like neuronal or glial p-tau, not as linear as threads
  - **White matter axonal threads:** threads of p-tau in white matter axons
- Glial inclusions:
  - **Gray matter / white matter astrocytic:** general astrocyte label, star shaped, includes astrocytic plaques (plaque-like clusters of astroglial p-tau), tufted/thorny astrocytes (compact and symmetrical perinuclear astroglial p-tau), ramified astrocytes (compact and asymmetric ramifications of perinuclear astroglial p-tau), and fuzzy granular astrocytes (fine-grained perinuclear astroglial p-tau)
  - **Gray matter / white matter coiled body:** circular or crescent shaped coiled body, oligodendrocytes are more common in white matter, very small, often associated with a cell and can be wrapped around a nucleus
  - **Globular glial tauopathy:** gray matter clusters of round globules and dots of astroglial p-tau
- Normal appearing and background classes:
  - **Healthy neuron:** healthy, non-tangle bearing neuron
  - **Non-tissue background:** slide background, white area
  - **Tissue:** normal appearing tissue, not a neuron
  - **Stain background:** non-specific background staining
  - **Artifact:** staining artifact, non-physiological
  - **Dirt:** dirt, unidentifiable floating objects over tissue, shadows from glass

**pTDP-43 Pathology Training Data**

Slides from 65 specimens across neuropathological diagnoses (including ALS, LATE, FTLD-TDP-43 types A, B, C, and E, and AD) across Thal, Braak, and CERAD stages were selected for annotation. Within each diagnostic group, in some specimens we selected random slides to annotate. In other randomly chosen specimens, we selected slides in disease-specific regions of interest. In the cases assigned to annotate regions of interest, ALS cases included spinal cord, motor cortex, and medulla sections. LATE cases included sections from the amygdala, hippocampus, and a random cortical region. Finally, in the AD group, the amygdala, hippocampus, and other randomly selected slides were annotated. Cases in the FTLD diagnostic group always had random slides selected for annotations. 187 1D3-stained sections were annotated in total.

**pTDP-43 Pathology Annotation Criteria**

The following criteria were developed as descriptions of each type of inclusion. Criteria were developed with expert pathologists before the initial training event and then reviewed and edited throughout the annotation period in consultation with experts to clarify issues for annotators. The bolded text indicates the name of the label. Note that the categories here do not correspond to the classes used in final training.

- Gray matter inclusions
  - Gray matter neuronal cytoplasmic inclusions (NCIs): small-to large neuronal inclusions, confluent morphology in most or all of the cytoplasm of a neuron (unlike a GFNI, which is more granular), morphologies vary depending on the neuron it occupies (similar to how p-tau tangles can differ by neuron shape), more common in cortical layers II-III but can be found in other layers. If any of the pTDP-43 inclusions are very dense, it’s likely this or an NII. Two subcategories are labelled:
    - **NCI-round:** round, dot-like, dense, or crescent-shaped.
    - **NCI-ring-like:** perinuclear, looks like a ring completely around the cell.
  - **Gray matter neuronal intranuclear inclusion (NII):** small intranuclear inclusions. Two basic morphologies include small round inclusions inside the neuron, or lenticular (cat-eye lens shaped) inclusions typically found in the center of perikaryal / somas, round intranuclear inclusions are more common than lentinculear inclusions. Most often found in cortical layers II-III, but sometimes found in lower layers.
  - **Gray matter short dystrophic neurites:** short threads of <20 microns, short, straight, or curved, rarely tortuous. More common in cortical layers I-III
  - **Gray matter long dystrophic neurites:** long threads of >20 microns, long, straight, can be curved or tortuous. Found in any cortical layer. More likely to be “rope-like” and thicker.
  - **Gray matter grains:** small, dot-like inclusions in neuropil. Found in any cortical layer, more common in certain regions, especially limbic areas.
- Gray matter (other)
  - **Gray matter vacuoles:** small to large spherical holes in neuropil, spongiosis. More common in cortical layers II-III, can be found in lower layers. Different from perivascular spaces, which have micro-vessels, and artifacts in tissue, which have holes that are more elongated and less round
- White matter inclusions:
  - **White matter oligodendroglial inclusions:** small oligodendroglial inclusions. Small, curvilinear or round inclusions predominantly found in white matter, may be found in lower cortical layers. Check the white matter, but also annotate if you see them in the gray matter. In the gray matter, these will be smaller than NCIs. Often crescent shaped around the cell because of the anatomy of oligodendrocytes.
  - **White matter threads:** short or long dystrophic neurites similar to gray matter dystrophic neurites but found between oligodendroglia in the white matter
- Normal appearing and background
  - **Tissue:** normal appearing tissue without neurons
  - **Dirt:** dirt on the slide
  - **Non-tissue background:** slide background, white area
  - **Artifact:** various unexpected areas other than dirt, such as large starburst-like brown stain
  - **Healthy neurons:** non pTDP-43-bearing neuron
- Non-specific Staining: category that includes non-pathological staining, background staining, and cross-reactivity
  - **Non-specific neuronal background staining:** neuronal, often pyramidal staining in the hippocampus and amygdala. Not pathological. Also common in lentiform nucleus and motor neurons. Fainter brown staining throughout the whole neuron, but no dark or dense inclusions
  - **Granulovacuolar degeneration (GVD):** similar to the background staining in hippocampus/amygdala/lentiform nucleus, but more granular with lots of small dark dots in the cell
  - **Neuromelanin:** staining found in the substantia nigra, pons, midbrain, and medulla. Grainy and tends to have a gray or greenish hue. Can be found intra- or extracellularly.

**Anatomical Sampling Regions of Interest**

Regions were annotated using anatomical landmarks and geometrical rules, as well as clearly visible patterns of cytoarchitecture, such as p-tau pathology in the transentorhinal region marking the transition from entorhinal cortex to BA35. These ROIs were selected to prioritize accuracy of the label rather than to maximize the area sampled. As a result, there are gaps between sampling regions in areas estimated to contain the borders between subfields. The MTL cortex in these hippocampal slides was labeled as entorhinal cortex and Brodmann areas 35 and 36. However, most histology sections were from the hippocampal body. In these sections, this cortex is likely more accurately described as parahippocampal cortex, specifically area TH. These regions were labeled as entorhinal cortex and BA35/36 for consistency with the CNDR’s neuropathology protocol, which typically uses the cortex on the hippocampal section as the sampling place for entorhinal cortex.

For each section, the estimated anatomical location of the slide along the longitudinal axis of the hippocampus (head, body, tail) was noted. Sections in the hippocampal tail (defined as slides where the typical hippocampal head or body shape was not present) were rare and not annotated. Areas of the slide with severe tearing or staining artifacts were not included in annotations. Multiple sampling regions were placed in a single anatomical ROI across the dentate gyrus and in areas of the cortex with tearing or curvature where a single rectangular box would cover only a small portion of the region.

**Extraction and Evaluation of ROI-level Quantitative Pathology Measures.**

Measures of pathology burden were generated in each anatomical ROI for tangles, threads, neuronal/glial pTDP-43, and neuritic pTDP-43. To derive these measures, first, the digital histology image region containing each sampling region was resampled to 0.4mm x 0.4mm resolution and input to the corresponding (p-tau or pTDP-43) *WildCat* model trained using the full available annotated data, yielding an attention heat map for each class. The heat map for each class was thresholded at zero, thus focusing on positive activation from the model. Each sampling region was broken up into contiguous segments of size ~200µm x 200µm using METIS, a graph partitioning algorithm [1]. For each segment, the average value of the thresholded heat map for each was computed. We performed experiments of different methods of aggregating these segment-level measures into a single sampling region-level pathology burden measure, including taking the mean, median, maximum, and various percentiles of the segment-level measurements. Validation with the semi-quantitative ratings given by an expert rater (p-tau: SA, pTDP-43: JR) was used to select the best aggregation method. For validation, measurements of segments within a single sampling box were aggregated. Mann-Whitney AUC tests were performed of the quantitative measures between adjacent semi-quantitative rating categories (i.e. 0 and 0.5). ANOVA linear contrasts were also performed across all ratings for each measurement, and the F-statistic was calculated. The 99^th^ percentile of the patch level measurements was ultimately chosen because it showed high AUC values and linear contrast F-statistics across all pathology types (Supplemental Table 3 and Supplemental Figures 1 and 2). As most anatomical regions were sampled across multiple sampling boxes, for all following analyses all segments for each anatomical ROI (e.g. CA1) were grouped and the 99^th^ percentile was taken as the ROI-level pathology measure.

**MR Scanning Acquisition Details**

MR scanning parameters varied as imaging was collected over the span of over two decades, so there were variations in field strength, scanner type, and scanning parameters. Field strength was either 1.5T (N = 48) or 3T (N = 115). A list of scanner models is provided below, with the number of participants scanned on each model:

- Siemens Prisma: 12
- Siemens Prisma Fit: 18
- Siemens Skyra: 2
- Siemens Sonata: 35
- Siemens Trio: 23
- Siemens TrioTim: 52
- Siemens Verio: 8
- GE Medical Systems, Genesis Signa: 8
- GE Medical Systems, Optima MR450w: 1
- GE Medical Systems, SIGNA EXCITE: 1
- GE Medical Systems, Signa HDxt: 1
- Siemens Espree: 1
- Siemens Symphony: 1

The median and range of primary acquisition parameters for 150 cases that used MPRAGE or similar sequences are given below:

- Echo time (ms): 3.090, (1.89-3.90)
- Repetition time (ms): 2300, (1620-3000)
- Flip angle (ms): 9, (8-15)
- Voxel output size (mm^3^): 0.954 (0.512-1.875)

The remaining 13 images were acquired on GE Medical Systems scanners and Siemens Espree and Symphony scanners using other kinds of MRI sequences with parameter values that are not comparable, and hence excluded from the summary values above.

**2. Supplemental Results**

| Label Name | Tangles | Threads | Background | Other | Total | Class | Accuracy |
| --- | --- | --- | --- | --- | --- | --- | --- |
| Neurofibrillary tangles | 86.41% | 6.68% | 2.23% | 4.69% | 1707 | Tangles | 86.41% |
| Non-Pyramidal tangle | 85.82% | 5.94% | 4.24% | 3.99% | 1178 | Tangles | 85.82% |
| Pretangle | 74.62% | 17.69% | 6.15% | 1.54% | 260 | Tangles | 74.62% |
| Gray matter threads | 3.16% | 86.60% | 6.23% | 4.00% | 1075 | Threads | 86.60% |
| Gray matter grains | 3.14% | 72.65% | 21.75% | 2.47% | 446 | Threads | 72.65% |
| Gray matter neuritic plaques | 16.18% | 58.90% | 4.85% | 20.06% | 309 | Threads | 58.90% |
| White matter axonal threads | 0.65% | 43.18% | 55.84% | 0.32% | 308 | Threads | 43.18% |
| Gray matter tangle associated neuritic cluster | 0.00% | 100.00% | 0.00% | 0.00% | 2 | Threads | 100.00% |
| Healthy neuron | 2.40% | 14.54% | 82.30% | 0.76% | 791 | Background | 82.30% |
| Non-tissue background | 0.13% | 1.61% | 98.25% | 0.00% | 744 | Background | 98.25% |
| Tissue | 0.43% | 6.46% | 92.40% | 0.72% | 697 | Background | 92.40% |
| Artifact | 1.96% | 8.57% | 88.57% | 0.89% | 560 | Background | 88.57% |
| Stain background | 2.23% | 15.03% | 81.82% | 0.93% | 539 | Background | 81.82% |
| Dirt | 6.39% | 14.43% | 78.14% | 1.03% | 485 | Background | 78.14% |
| Astrocytic p-tau | 5.14% | 28.98% | 3.76% | 62.12% | 1011 | Other | 62.12% |
| Coiled body | 47.55% | 35.66% | 10.49% | 6.29% | 143 | Other | 6.29% |
| Gray matter ballooned neuron | 87.23% | 4.26% | 6.38% | 2.13% | 47 | Other | 2.13% |
| Globular Glial Tauopathy | 50.00% | 0.00% | 0.00% | 50.00% | 2 | Other | 50.00% |

**Supplementary Table 1: Five-fold cross validation for *WildCat* training of p-tau inclusions for each individual label.** Given is the percentage of labels assigned to each class by *WildCat* during five-fold cross validation, the total number of annotations for each label, true class, and accuracy of prediction of *WildCat* to the correct class

| Label Name | Neuronal/Glial | Neuritic | Background | Non-Specific | Total | Class | Accuracy |
| --- | --- | --- | --- | --- | --- | --- | --- |
| Round neuronal cytoplasmic inclusions | 94.58% | 4.13% | 0.58% | 0.71% | 1551 | Neuronal | 94.58% |
| White matter oligodendroglial inclusions | 90.75% | 6.72% | 2.39% | 0.15% | 670 | Neuronal | 90.75% |
| Ring-like neuronal cytoplasmic inclusions | 89.60% | 5.94% | 0.99% | 3.47% | 202 | Neuronal | 89.60% |
| Neuronal nuclear inclusions | 68.29% | 26.83% | 2.44% | 2.44% | 41 | Neuronal | 68.29% |
| Short dystrophic neurites | 3.33% | 95.58% | 0.48% | 0.62% | 2102 | Neuritic | 95.58% |
| Long dystrophic neurites | 1.44% | 98.32% | 0.00% | 0.24% | 416 | Neuritic | 98.32% |
| Grains | 2.53% | 91.16% | 4.29% | 2.02% | 396 | Neuritic | 91.16% |
| White matter threads | 5.30% | 94.39% | 0.00% | 0.31% | 321 | Neuritic | 94.39% |
| Non-specific staining | 1.24% | 0.67% | 8.66% | 89.43% | 889 | Non-specific | 89.43% |
| Neuromelanin | 1.17% | 0.00% | 0.91% | 97.91% | 766 | Non-specific | 97.91% |
| Granulovacuolar degeneration | 3.13% | 4.17% | 3.13% | 89.58% | 96 | Non-specific | 89.58% |
| Tissue | 0.32% | 2.88% | 95.60% | 1.20% | 1249 | Background | 95.60% |
| Healthy neuron | 1.07% | 3.01% | 89.72% | 6.21% | 1031 | Background | 89.72% |
| Non-tissue background | 0.00% | 0.21% | 99.79% | 0.00% | 969 | Background | 99.79% |
| Dirt | 8.41% | 5.11% | 83.33% | 3.15% | 666 | Background | 83.33% |
| Artifact | 0.34% | 3.23% | 81.32% | 15.11% | 589 | Background | 81.32% |
| Vacuoles | 1.12% | 3.55% | 88.79% | 6.54% | 535 | Background | 88.79% |

**Supplementary Table 2: Five-fold cross validation for *WildCat* training of pTDP-43 inclusions for each individual label.** Given is the percentage of labels assigned to each class by *WildCat* during five-fold cross validation, the total number of annotations for each label, true class, and accuracy of prediction of *WildCat* to the correct class

| **Tangles** | | | | | | | | | | |
| --- | --- | --- | --- | --- | --- | --- | --- | --- | --- | --- |
|  | 0 vs. 0.5 | | 0.5 vs 1 | | 1 vs. 2 | | 2 vs. 3 | | Linear Contrast | |
|  | AUC | p | AUC | p | AUC | p | AUC | p | F | p |
| Mean | 0.796 | 0.050 | 0.917 | 0.015 | 0.800 | 0.008 | 0.805 | 0.003 | 64.24 | 5.16E-11 |
| Median | 0.444 | 0.755 | 0.625 | 0.205 | 0.754 | 0.015 | 0.788 | 0.006 | 40.27 | 3.42E-08 |
| Maximum | 0.944 | 0.006 | 0.722 | 0.147 | 0.592 | 0.248 | 0.695 | 0.044 | 82.82 | 7.76E-13 |
| 25^th^ Percentile | 0.444 | 0.755 | 0.542 | 0.369 | 0.658 | 0.048 | 0.733 | 0.019 | 13.37 | 5.46E-04 |
| 75^th^ Percentile | 0.417 | 0.795 | 0.792 | 0.059 | 0.846 | 0.003 | 0.838 | 9.41E-04 | 96.50 | 4.99E-14 |
| 90^th^ Percentile | 0.556 | 0.387 | 0.875 | 0.030 | 0.775 | 0.015 | 0.800 | 0.003 | 75.31 | 3.94E-12 |
| 95^th^ Percentile | 0.639 | 0.218 | 0.917 | 0.015 | 0.708 | 0.054 | 0.762 | 0.010 | 79.77 | 1.49E-12 |
| 99^th^ Percentile | 0.926 | 0.008 | 0.806 | 0.068 | 0.650 | 0.127 | 0.690 | 0.048 | 79.34 | 1.63E-12 |
| **Threads** | | | | | | | | | | |
|  | 0 vs. 0.5 | | 0.5 vs 1 | | 1 vs. 2 | | 2 vs. 3 | | Linear Contrast | |
|  | AUC | p | AUC | p | AUC | p | AUC | p | F | p |
| Mean | 0.889 | 0.025 | 0.852 | 0.031 | 0.748 | 0.007 | 0.446 | 0.701 | 5.17 | 0.027 |
| Median | 0.861 | 0.031 | 0.833 | 0.040 | 0.737 | 0.010 | 0.442 | 0.714 | 4.54 | 0.037 |
| Maximum | 0.889 | 0.025 | 0.833 | 0.040 | 0.715 | 0.018 | 0.496 | 0.523 | 9.60 | 0.003 |
| 25^th^ Percentile | 0.722 | 0.111 | 0.852 | 0.031 | 0.693 | 0.031 | 0.450 | 0.687 | 3.34 | 0.073 |
| 75^th^ Percentile | 0.889 | 0.024 | 0.815 | 0.050 | 0.759 | 0.005 | 0.438 | 0.727 | 5.84 | 0.019 |
| 90^th^ Percentile | 0.889 | 0.025 | 0.833 | 0.040 | 0.726 | 0.014 | 0.438 | 0.727 | 6.74 | 0.012 |
| 95^th^ Percentile | 0.889 | 0.025 | 0.833 | 0.040 | 0.733 | 0.011 | 0.467 | 0.630 | 7.53 | 0.008 |
| 99^th^ Percentile | 0.889 | 0.025 | 0.833 | 0.040 | 0.722 | 0.015 | 0.475 | 0.600 | 9.08 | 0.004 |
| **Neuronal/Glial pTDP-43** | | | | | | | | | | |
|  | 0 vs. 0.5 | | 0.5 vs 1 | | 1 vs. 2 | | 2 vs. 3 | | Linear Contrast | |
|  | AUC | p | AUC | p | AUC | p | AUC | p | F | p |
| Mean | 0.793 | 0.004 | 0.698 | 0.052 | 0.870 | 3.23E-04 | 0.489 | 0.551 | 35.36 | 5.42E-08 |
| Median | 0.588 | 0.076 | 0.526 | 0.406 | 0.843 | 6.94E-04 | 0.422 | 0.697 | 18.39 | 4.61E-05 |
| Maximum | 0.755 | 0.011 | 0.766 | 0.013 | 0.792 | 0.005 | 0.689 | 0.149 | 142.81 | 4.01E-20 |
| 25^th^ Percentile | 0.553 | 0.076 | 0.497 | 0.530 | 0.775 | 0.001 | 0.289 | 0.923 | 10.71 | 0.002 |
| 75^th^ Percentile | 0.657 | 0.034 | 0.630 | 0.140 | 0.866 | 7.30E-04 | 0.467 | 0.601 | 52.25 | 1.69E-10 |
| 90^th^ Percentile | 0.673 | 0.054 | 0.688 | 0.061 | 0.861 | 8.63E-04 | 0.556 | 0.399 | 101.04 | 2.82E-16 |
| 95^th^ Percentile | 0.787 | 0.005 | 0.729 | 0.029 | 0.861 | 4.69E-04 | 0.511 | 0.500 | 144.97 | 2.66E-20 |
| 99^th^ Percentile | 0.769 | 0.008 | 0.760 | 0.015 | 0.838 | 0.001 | 0.622 | 0.259 | 158.97 | 2.00E-21 |
| **Neuritic pTDP-43** | | | | | | | | | | |
|  | 0 vs. 0.5 | | 0.5 vs 1 | | 1 vs. 2 | | 2 vs. 3 | | Linear Contrast | |
|  | AUC | p | AUC | p | AUC | p | AUC | p | F | p |
| Mean | 0.844 | 9.01E-06 | 0.640 | 0.055 | 0.928 | 3.11E-06 | 0.694 | 0.182 | 133.18 | 2.65E-19 |
| Median | 0.749 | 4.79E-04 | 0.595 | 0.139 | 0.899 | 7.00E-05 | 0.583 | 0.367 | 86.89 | 8.94E-15 |
| Maximum | 0.843 | 9.74E-06 | 0.713 | 0.007 | 0.931 | 2.41E-06 | 0.750 | 0.117 | 180.03 | 5.37E-23 |
| 25^th^ Percentile | 0.672 | 0.005 | 0.565 | 0.221 | 0.870 | 1.94E-04 | 0.556 | 0.420 | 36.36 | 3.77E-08 |
| 75^th^ Percentile | 0.817 | 3.14E-05 | 0.652 | 0.041 | 0.935 | 1.86E-06 | 0.639 | 0.268 | 154.60 | 4.42E-21 |
| 90^th^ Percentile | 0.864 | 2.86E-06 | 0.648 | 0.045 | 0.949 | 6.05E-07 | 0.750 | 0.117 | 191.81 | 8.01E-24 |
| 95^th^ Percentile | 0.860 | 3.73E-06 | 0.670 | 0.026 | 0.942 | 1.08E-06 | 0.750 | 0.117 | 191.88 | 7.92E-24 |
| 99^th^ Percentile | 0.850 | 6.60E-06 | 0.708 | 0.008 | 0.938 | 1.42E-06 | 0.722 | 0.147 | 192.56 | 7.11E-24 |

**Supplementary Table 3: Comparison of different methods of generating quantitative pathology measurements across expert ratings.** Each region was rated separately for tangles and threads or neuronal/glial and neuritic pTDP-43 from 0-3 by an expert rater blind to all quantitative data. Segment level measurements within a single sampling box were aggregated to form quantitative data by a variety of methods, including taking the mean activation, median, maximum, and a variety of percentiles. To select the best method, Mann Whitney AUC tests were performed for each measurement between adjacent categories (i.e. 0 and 0.5**).**  ANOVA linear contrasts were also performed across all ratings for each measurement; the F statistic and p-value for this test is shown at the right of each row. The 99^th^ percentile was ultimately chosen because it showed high AUC values and linear contrast F statistics across all pathology types

|  | **Quantitative Tau and Imaging** | | | | **Quantitative TDP-43 and Imaging** | | | | **Quantitative Tau, TDP-43, and Imaging** | | | |
| --- | --- | --- | --- | --- | --- | --- | --- | --- | --- | --- | --- | --- |
|  | **Demographics** | | | | | | | | | | | |
| N | 140 | | | | 142 | | | | 126 | | | |
| Age | 74.57 $\pm$ 10.19, 51-101 | | | | 75.06 $\pm$ 9.94, 51-101 | | | | 74.88 $\pm$ 10.19, 51-101 | | | |
| Sex | 92 M / 48 F | | | | 95 M / 47 F | | | | 81 M / 45 F | | | |
|  | **Neuropathological Diagnoses** | | | | | | | | | | | |
|  | Primary Diagnosis | | Secondary Diagnosis | | Primary Diagnosis | | Secondary Diagnosis | | Primary Diagnosis | | Secondary Diagnosis | |
| Alzheimer’s disease | 89 | | 37 | | 87 | | 40 | | 77 | | 35 | |
| Lewy Body disease | 46 | | 42 | | 50 | | 40 | | 44 | | 34 | |
| Cerebrovascular disease | 4 | | 5 | | 4 | | 5 | | 4 | | 5 | |
| LATE | 1 | | 14 | | 1 | | 15 | | 1 | | 12 | |
| Primary age-related tauopathy | 0 | | 10 | | 0 | | 10 | | 0 | | 10 | |
| Cerebral amyloid angiopathy | 0 | | 5 | | 0 | | 6 | | 0 | | 5 | |
| Hippocampal Sclerosis | 0 | | 2 | | 0 | | 2 | | 0 | | 2 | |
|  | **Neuropathological Staging** | | | | | | | | | | | |
|  | 0 | 1 | 2 | 3 | 0 | 1 | 2 | 3 | 0 | 1 | 2 | 3 |
| A | 12 | 13 | 19 | 96 | 13 | 12 | 21 | 96 | 12 | 11 | 19 | 84 |
| B | 3 | 26 | 26 | 85 | 4 | 28 | 24 | 86 | 3 | 26 | 22 | 75 |
| C | 22 | 14 | 20 | 84 | 26 | 14 | 19 | 83 | 22 | 14 | 18 | 72 |
| LATE-NC | 109 | 17 | 12 | 2 | 113 | 15 | 12 | 2 | 102 | 14 | 8 | 2 |

**Supplementary Table 4: Demographics for cases with quantitative p-tau data and ipsilateral imaging, quantitative pTDP-43 data and ipsilateral imaging, and both quantitative pathology measures and ipsilateral imaging.** This demographics table contains the intersections from table 1 of the main text. The “Quantitative Tau and Imaging” was included in model comparisons and the main analyses and consists of all cases with quantitative p-tau summary pathology measures and imaging of the ipsilateral hemisphere passing segmentation quality control. The “Quantitative TDP-43 and Imaging” cohort was included in model comparisons only and consists of the cases with quantitative pTDP-43 summary pathology measures and imaging of the ipsilateral hemisphere passing segmentation quality control. The “Quantitative Tau, TDP-43, and Imaging” cohort was included in Figure 6 and in supplemental analyses where all four quantitative summary pathology measures were analyzed in relation to structure and consists of all cases with both quantitative p-tau and pTDP-43 summary measures and imaging of the ipsilateral hemisphere passing segmentation quality control. A = Amyloid, B = Braak, C = CERAD, LATE-NC = Limbic predominant age-related TDP-43 encephalopathy neuropathological change

|  | AH (vol) | PH (vol) | ERC (thk) | BA35 (thk) | BA36 (thk) | PHC (thk) |
| --- | --- | --- | --- | --- | --- | --- |
| LATE-NC Stage 1 (N = 17, df = 8/9) | | | | | | |
| Tangles (Q) | *β = -0.50,*  *t = -2.05,*  *p = 0.037* | β = -0.39,  t = -0.88,  p = 0.202 | *β = -0.58,*  *t = -2.21,*  *p = 0.027* | β = -0.16,  t = -0.78,  p = 0.227 | β = 0.36,  t = 0.79,  p = 0.776 | β = 0.03,  t = 0.08,  p = 0.532 |
| Threads (Q) | β = 0.09,  t = 0.43,  p = 0.662 | β = 0.33,  t = 0.91,  p = 0.805 | β = 0.35,  t = 1.55,  p = 0.923 | *β = -0.51,*  *t = -2.94,*  *p = 0.008* | β = -0.50,  t = -1.31,  p = 0.112 | β = -0.34,  t = -1.32,  p = 0.109 |
| MTL pTDP-43 (SQ) | β = 0.15,  t = 0.79,  p = 0.773 | β = 0.26,  t = 0.77,  p = 0.767 | β = -0.22,  t = -1.05,  p = 0.160 | β = 0.14,  t = 0.83,  p = 0.787 | β = -0.20,  t = -0.53,  p = 0.303 | β = -0.42,  t = -1.74,  p = 0.058 |
| LATE-NC Stage 2/3 (N = 14, df = 5/6) | | | | | | |
| Tangles (Q) | β = -0.54,  t = -1.09,  p = 0.162 | β = -0.08,  t = -0.15,  p = 0.442 | β = -0.21,  t = -0.33,  p = 0.376 | β = -0.15,  t = -0.25,  p = 0.406 | β = -0.94,  t = -1.90,  p = 0.053 | β = -0.27,  t = -0.48,  p = 0.326 |
| Threads (Q) | β = 0.68,  t = 1.71,  p = 0.926 | β = 0.08,  t = 0.19,  p = 0.570 | β = -0.11,  t = -0.21,  p = 0.421 | β = 0.64,  t = 1.31,  p = 0.882 | β = 0.82,  t = 2.02,  p = 0.955 | β = 0.35,  t = 0.77,  p = 0.766 |
| MTL pTDP-43 (SQ) | β = -0.24,  t = -0.56,  p = 0.298 | β = 0.36,  t = 0.80,  p = 0.769 | β = -0.12,  t = -0.22,  p = 0.418 | β = 0.36,  t = 0.69,  p = 0.742 | β = -0.34,  t = -0.78,  p = 0.232 | β = -0.45,  t = -0.93,  p = 0.195 |

**Supplementary Table 5: Pathology to structure analyses in cases with quantitative p-tau pathology measures and ipsilateral MRI, divided by LATE staging.** The standardized β coefficient, t statistic, and p value for the association of each pathology measure with each imaging ROI is given within each cohort (top: cases with LATE stage 1, N = 17, bottom: cases with LATE stage 2 or 3, N = 14). Degrees of freedom (df) are given for each cohort; the first number is for AH/PH associations due to the additional covariate of ICV, while the second number is for ERC, BA35, BA36, and PHC associations. Associations that would survive Bonferroni correction (P < 8.33e-03) are bolded; associations that are significant at P < 0.05 but would not survive corrections are italicized. AH = Anterior hippocampus volume, PH = posterior hippocampus volume, ERC = entorhinal cortex median thickness, BA35 = Brodmann area 35 median thickness, BA36 = Brodmann area 36 median thickness, PHC = parahippocampal cortex median thickness. Tangles (Q) = quantitative tangles summary measure, Threads (Q) = quantitative threads summary measure, MTL pTDP-43 = semi-quantitative MTL pTDP43 rating

|  | AH (vol) | PH (vol) | ERC (thk) | BA35 (thk) | BA36 (thk) | PHC (thk) |
| --- | --- | --- | --- | --- | --- | --- |
| Quantitative Tau and TDP-43 Parameters (df = 116/117) | | | | | | |
| Tangles (Q) | **β = -0.28,**  **t = -3.30,**  **p = 6.34e-04** | **β = -0.41,**  **t = -4.96,**  **p = 1.22e-06** | **β = -0.44,**  **t = -4.95,**  **p = 1.27e-06** | **β = -0.43,**  **t = -4.52,**  **p = 7.46e-06** | **β = -0.26,**  **t = -2.57,**  **p = 0.006** | **β = -0.30,**  **t = -3.08,**  **p = 0.001** |
| Threads (Q) | β = -0.11,  t = -1.25,  p = 0.107 | β = -0.03,  t = -0.34,  p = 0.366 | β = 0.00,  t = 0.01,  p = 0.502 | β = 0.12,  t = 1.33,  p = 0.908 | β = -0.07,  t = -0.72,  p = 0.235 | β = -0.02,  t = -0.19,  p = 0.425 |
| Neuronal/Glial pTDP-43 (Q) | **β = -0.21,**  **t = -2.45,**  **p = 0.008** | **β = -0.21,**  **t = -2.51,**  **p = 0.007** | *β = -0.17,*  *t = -1.84,*  *p = 0.034* | *β = -0.16,*  *t = -1.71,*  *p = 0.045* | β = -0.07,  t = -0.69,  p = 0.244 | *β = -0.16,*  *t = -1.68,*  *p = 0.048* |
| Neuritic pTDP-43 (Q) | β = 0.15,  t = 1.80,  p = 0.963 | β = 0.06,  t = 0.74,  p = 0.769 | β = -0.09,  t = -1.00,  p = 0.161 | β = 0.21,  t = 2.28,  p = 0.988 | β = 0.05,  t = 0.48,  p = 0.682 | β = 0.07,  t = 0.70,  p = 0.757 |
| Quantitative Tau and Semi-Quantitative TDP-43 Parameters (df = 117, 118) | | | | | | |
| Tangles (Q) | **β = -0.29,**  **t = -3.48,**  **p = 3.56e-04** | **β = -0.43,**  **t = -5.64,**  **p = 6.10e-08** | **β = -0.49,**  **t = -5.63,**  **p = 6.24e-08** | **β = -0.42,**  **t = -4.38,**  **p = 1.28e-05** | **β = -0.26,**  **t = -2.68,**  **p = 0.004** | **β = -0.31,**  **t = -3.29,**  **p = 6.55e-04** |
| Threads (Q) | β = -0.09,  t = -1.03,  p = 0.153 | β = -0.01,  t = -0.13,  p = 0.449 | β = 0.02,  t = 0.25,  p = 0.600 | β = 0.15,  t = 1.61,  p = 0.945 | β = -0.06,  t = -0.67,  p = 0.254 | β = 0.01,  t = 0.09,  p = 0.534 |
| MTL pTDP-43 (SQ) | **β = -0.22,**  **t = -2.88,**  **p = 0.002** | **β = -0.30,**  **t = -4.29,**  **p = 1.84e-05** | **β = -0.22,**  **t = -2.78,**  **p = 0.003** | β = 0.01,  t = 0.14,  p = 0.555 | β = -0.09,  t = -0.95,  p = 0.172 | β = -0.06,  t = -0.74,  p = 0.230 |
| Semi-Quantitative Tau and TDP-43 Parameters (df = 118, 119) | | | | | | |
| MTL p-Tau (SQ) | β = -0.12,  t = -1.41,  p = 0.081 | **β = -0.28,**  **t = -3.59,**  **p = 2.45e-04** | **β = -0.34,**  **t = -3.85,**  **p = 9.68e-05** | **β = -0.23,**  **t = -2.44,**  **p = 0.008** | **β = -0.24,**  **t = -2.59,**  **p = 0.005** | **β = -0.27,**  **t = -2.98,**  **p = 0.002** |
| MTL pTDP-43 (SQ) | *β = -0.19,*  *t = -2.28,*  *p = 0.012* | **β = -0.24,**  **t = -3.12,**  **p = 0.001** | *β = -0.15,*  *t = -1.76,*  *p = 0.040* | β = 0.05,  t = 0.56,  p = 0.713 | β = -0.03,  t = -0.37,  p = 0.357 | β = -0.01,  t = -0.13,  p = 0.449 |

**Supplementary Table 6: Pathology to structure analyses in cases with both quantitative pathology measures and ipsilateral MRI (N = 126).** Each section represents a different linear model (top: quantitative p-tau and pTDP-43 parameters used; middle: quantitative p-tau and semi-quantitative pTDP-43 measures used, as in the main text; bottom: semi-quantitative p-tau and pTDP-43 measures used). All models were covaried for age, antemortem interval, MRI field strength, and sex; models for AH and PH also included ICV as a covariate. Degrees of freedom (df) are given for each model; the first number is for AH/PH associations due to the additional covariate of ICV, while the second number is for ERC, BA35, BA36, and PHC associations. The standardized β coefficient, t statistic, and p value for the association of each pathology measure with each imaging ROI is given within each model. Associations that would survive Bonferroni correction (P < 8.33e-03) are bolded; associations that are significant at P < 0.05 but would not survive corrections are italicized. AH = Anterior hippocampus volume, PH = posterior hippocampus volume, ERC = entorhinal cortex median thickness, BA35 = Brodmann area 35 median thickness, BA36 = Brodmann area 36 median thickness, PHC = parahippocampal cortex median thickness. Tangles (Q) = quantitative tangles summary measure, Threads (Q) = quantitative threads summary measure, Neuronal/Glial pTDP-43 (Q) = quantitative neuronal/glial pTDP-43 summary measure, Neuritic pTDP-43 (Q) = quantitative neuritic pTDP-43 summary measure, MTL pTDP-43 = semi-quantitative MTL pTDP43 rating, MTL p-Tau (SQ) = semi-quantitative MTL p-Tau rating

|  | AH (vol) | PH (vol) | ERC (thk) | BA35 (thk) | BA36 (thk) | PHC (thk) |
| --- | --- | --- | --- | --- | --- | --- |
| Quantitative Tau and TDP-43 Parameters (df = 14/15) | | | | | | |
| Tangles (Q) | β = -0.22,  t = -1.12,  p = 0.142 | β = 0.14,  t = 0.48,  p = 0.681 | *β = -0.44,*  *t = -2.39,*  *p = 0.015* | β = -0.33,  t = -1.29,  p = 0.109 | β = -0.19,  t = -0.77,  p = 0.228 | β = -0.30,  t = -1.21,  p = 0.122 |
| Threads (Q) | β = 0.05,  t = 0.28,  p = 0.607 | β = -0.06,  t = -0.19,  p = 0.426 | β = 0.27,  t = 1.34,  p = 0.900 | β = 0.01,  t = 0.03,  p = 0.514 | β = 0.17,  t = 0.61,  p = 0.724 | β = -0.01,  t = -0.04,  p = 0.483 |
| Neuronal/Glial pTDP-43 (Q) | *β = -0.40,*  *t = -2.48,*  *p = 0.013* | *β = -0.55,*  *t = -2.31,*  *p = 0.018* | β = -0.45,  t = -2.70,  p = 0.008 | β = -0.36,  t = -1.58,  p = 0.067 | β = -0.28,  t = -1.24,  p = 0.116 | β = -0.28,  t = -1.27,  p = 0.111 |
| Neuritic pTDP-43 (Q) | β = 0.16,  t = 0.97,  p = 0.826 | β = 0.02,  t = 0.09,  p = 0.535 | *β = -0.39,*  *t = -2.36,*  *p = 0.016* | β = 0.38,  t = 1.65,  p = 0.940 | β = 0.01,  t = 0.05,  p = 0.520 | β = 0.06,  t = 0.27,  p = 0.603 |
| Quantitative Tau and Semi-Quantitative TDP-43 Parameters (df = 15/16) | | | | | | |
| Tangles (Q) | *β = -0.40,*  *t = -2.07,*  *p = 0.028* | β = -0.14,  t = -0.54,  p = 0.298 | **β = -0.62,**  **t = -3.10,**  **p = 0.003** | β = -0.43,  t = -1.54,  p = 0.071 | β = -0.31,  t = -1.25,  p = 0.115 | β = -0.40,  t = -1.62,  p = 0.063 |
| Threads (Q) | β = 0.26,  t = 1.59,  p = 0.934 | β = 0.13,  t = 0.59,  p = 0.717 | β = 0.19,  t = 1.01,  p = 0.836 | β = 0.29,  t = 1.13,  p = 0.862 | β = 0.25,  t = 1.10,  p = 0.857 | β = 0.09,  t = 0.41,  p = 0.656 |
| MTL pTDP-43 (SQ) | *β = -0.40,*  *t = -2.63,*  *p = 0.009* | **β = -0.62,**  **t = -3.05,**  **p = 0.004** | **β = -0.56,**  **t = -3.25,**  **p = 0.003** | β = -0.05,  t = -0.22,  p = 0.413 | β = -0.26,  t = -1.26,  p = 0.112 | β = -0.19,  t = -0.91,  p = 0.189 |
| Semi-Quantitative Tau and TDP-43 Parameters (df = 16/17) | | | | | | |
| MTL p-Tau (SQ) | β = -0.13,  t = -0.92,  p = 0.185 | β = 0.06,  t = 0.33,  p = 0.626 | β = -0.22,  t = -1.25,  p = 0.114 | β = 0.00,  t = 0.00,  p = 0.501 | β = -0.19,  t = -1.03,  p = 0.158 | *β = -0.37,*  *t = -2.14,*  *p = 0.024* |
| MTL pTDP-43 (SQ) | *β = -0.33,*  *t = -2.06,*  *p = 0.028* | **β = -0.60,**  **t = -3.06,**  **p = 0.004** | *β = -0.45,*  *t = -2.30,*  *p = 0.017* | β = 0.02,  t = 0.09,  p = 0.536 | β = -0.22,  t = -1.06,  p = 0.151 | β = -0.13,  t = -0.68,  p = 0.252 |

**Supplementary Table 7: Pathology to structure analyses in cases with both quantitative pathology measures, ipsilateral MRI, and LATE-NC stages 1-3, (N = 24).** Each section represents a different linear model (top: quantitative p-tau and pTDP-43 parameters used; middle: quantitative p-tau and semi-quantitative pTDP-43 measures used, as in the main text; bottom: semi-quantitative p-tau and pTDP-43 measures used). All models were covaried for age, antemortem interval, MRI field strength, and sex; models for AH and PH also included ICV as a covariate. Degrees of freedom (df) are given for each model; the first number is for AH/PH associations due to the additional covariate of ICV, while the second number is for ERC, BA35, BA36, and PHC associations. The standardized β coefficient, t statistic, and p value for the association of each pathology measure with each imaging ROI is given within each model. Associations that would survive Bonferroni correction (P < 8.33e-03) are bolded; associations that are significant at P < 0.05 but would not survive corrections are italicized. AH = Anterior hippocampus volume, PH = posterior hippocampus volume, ERC = entorhinal cortex median thickness, BA35 = Brodmann area 35 median thickness, BA36 = Brodmann area 36 median thickness, PHC = parahippocampal cortex median thickness. Tangles (Q) = quantitative tangles summary measure, Threads (Q) = quantitative threads summary measure, Neuronal/Glial pTDP-43 (Q) = quantitative neuronal/glial pTDP-43 summary measure, Neuritic pTDP-43 (Q) = quantitative neuritic pTDP-43 summary measure, MTL pTDP-43 = semi-quantitative MTL pTDP43 rating, MTL p-Tau (SQ) = semi-quantitative MTL p-Tau rating

|  | AH (vol) | PH (vol) | ERC (thk) | BA35 (thk) | BA36 (thk) | PHC (thk) |
| --- | --- | --- | --- | --- | --- | --- |
| Quantitative Tau and TDP-43 Parameters (df = 65/66) | | | | | | |
| Tangles (Q) | **β = -0.33,**  **t = -3.36,**  **p = 6.47e-04** | **β = -0.51,**  **t = -5.27,**  **p = 8.3e-07** | **β = -0.44,**  **t = -3.86,**  **p = 1.32e-04** | **β = -0.41,**  **t = -3.68,**  **p = 2.33e-04** | β = -0.20,  t = -1.64,  p = 0.052 | β = -0.21,  t = -1.64,  p = 0.053 |
| Threads (Q) | *β = -0.20,*  *t = -1.95,*  *p = 0.028* | β = -0.02,  t = -0.22,  p = 0.414 | β = -0.06,  t = -0.54,  p = 0.294 | β = 0.09,  t = 0.80,  p = 0.786 | β = -0.14,  t = -1.14,  p = 0.130 | β = 0.00,  t = -0.02,  p = 0.490 |
| Neuronal/Glial pTDP-43 (Q) | *β = -0.20,*  *t = -1.81,*  *p = 0.038* | β = -0.15,  t = -1.39,  p = 0.085 | β = -0.18,  t = -1.47,  p = 0.073 | β = -0.09,  t = -0.74,  p = 0.231 | β = -0.06,  t = -0.48,  p = 0.318 | β = -0.16,  t = -1.14,  p = 0.130 |
| Neuritic pTDP-43 (Q) | β = 0.21,  t = 2.10,  p = 0.980 | β = 0.03,  t = 0.31,  p = 0.619 | β = -0.09,  t = -0.74,  p = 0.232 | β = 0.28,  t = 2.41,  p = 0.991 | β = 0.04,  t = 0.28,  p = 0.609 | β = 0.11,  t = 0.83,  p = 0.795 |
| Quantitative Tau and Semi-Quantitative TDP-43 Parameters (df = 66/67) | | | | | | |
| Tangles (Q) | **β = -0.37,**  **t = -3.66,**  **p = 2.54e-04** | **β = -0.55,**  **t = -6.01,**  **p = 4.39e-08** | **β = -0.48,**  **t = -4.27,**  **p = 3.19e-05** | **β = -0.39,**  **t = -3.39,**  **p = 5.92e-04** | *β = -0.21,*  *t = -1.69,*  *p = 0.048* | *β = -0.22,*  *t = -1.71,*  *p = 0.046* |
| Threads (Q) | **β = -0.18,**  **t = -1.73,**  **p = 0.044** | β = -0.01,  t = -0.07,  p = 0.474 | β = -0.04,  t = -0.36,  p = 0.359 | β = 0.11,  t = 0.96,  p = 0.830 | β = -0.13,  t = -1.08,  p = 0.142 | β = 0.02,  t = 0.18,  p = 0.569 |
| MTL pTDP-43 (SQ) | *β = -0.22,*  *t = -2.16,*  *p = 0.017* | **β = -0.28,**  **t = -3.01,**  **p = 0.002** | *β = -0.25,*  *t = -2.21,*  *p = 0.015* | β = 0.10,  t = 0.83,  p = 0.794 | β = -0.03,  t = -0.26,  p = 0.396 | β = -0.06,  t = -0.46,  p = 0.323 |
| Semi-Quantitative Tau and TDP-43 Parameters (df = 67/68) | | | | | | |
| MTL p-Tau (SQ) | β = -0.05,  t = -0.46,  p = 0.323 | *β = -0.25,*  *t = -2.25,*  *p = 0.014* | **β = -0.31,**  **t = -2.51,**  **p = 0.007** | β = -0.09,  t = -0.75,  p = 0.229 | β = -0.14,  t = -1.07,  p = 0.145 | β = -0.10,  t = -0.74,  p = 0.230 |
| MTL pTDP-43 (SQ) | β = -0.13,  t = -1.18,  p = 0.121 | β = -0.14,  t = -1.25,  p = 0.108 | β = -0.11,  t = -0.90,  p = 0.187 | β = 0.17,  t = 1.36,  p = 0.911 | β = 0.04,  t = 0.28,  p = 0.610 | β = -0.01,  t = -0.05,  p = 0.479 |

**Supplementary Table 8: Pathology to structure analyses in cases with both quantitative pathology measures, ipsilateral MRI, and Braak stage V/VI, (N = 75).** Each section represents a different linear model (top: quantitative p-tau and pTDP-43 parameters used; middle: quantitative p-tau and semi-quantitative pTDP-43 measures used, as in the main text; bottom: semi-quantitative p-tau and pTDP-43 measures used). All models were covaried for age, antemortem interval, MRI field strength, and sex; models for AH and PH also included ICV as a covariate. Degrees of freedom (df) are given for each model; the first number is for AH/PH associations due to the additional covariate of ICV, while the second number is for ERC, BA35, BA36, and PHC associations. The standardized β coefficient, t statistic, and p value for the association of each pathology measure with each imaging ROI is given within each model. Associations that would survive Bonferroni correction (P < 8.33e-03) are bolded; associations that are significant at P < 0.05 but would not survive corrections are italicized. AH = Anterior hippocampus volume, PH = posterior hippocampus volume, ERC = entorhinal cortex median thickness, BA35 = Brodmann area 35 median thickness, BA36 = Brodmann area 36 median thickness, PHC = parahippocampal cortex median thickness. Tangles (Q) = quantitative tangles summary measure, Threads (Q) = quantitative threads summary measure, Neuronal/Glial pTDP-43 (Q) = quantitative neuronal/glial pTDP-43 summary measure, Neuritic pTDP-43 (Q) = quantitative neuritic pTDP-43 summary measure, MTL pTDP-43 = semi-quantitative MTL pTDP43 rating, MTL p-Tau (SQ) = semi-quantitative MTL p-Tau rating

|  | AH (vol) | PH (vol) | ERC (thk) | BA35 (thk) | BA36 (thk) | PHC (thk) |
| --- | --- | --- | --- | --- | --- | --- |
| Whole Cohort (N = 51, df = 43/44) | | | | | | |
| Tangles (Q) | *β = -0.24,*  *t = -1.77,*  *p = 0.042* | **β = -0.37,**  **t = -2.85,**  **p = 0.003** | **β = -0.38,**  **t = -2.91,**  **p = 0.003** | **β = -0.36,**  **t = -2.64,**  **p = 0.006** | β = -0.09,  t = -0.70,  p = 0.243 | β = -0.10,  t = -0.66,  p = 0.256 |
| Threads (Q) | β = 0.06,  t = 0.41,  p = 0.659 | β = 0.03,  t = 0.19,  p = 0.574 | β = -0.06,  t = -0.45,  p = 0.326 | β = 0.06,  t = 0.46,  p = 0.676 | *β = -0.25,*  *t = -1.88,*  *p = 0.033* | β = -0.08,  t = -0.53,  p = 0.299 |
| MTL pTDP-43 (SQ) | *β = -0.25,*  *t = -1.88,*  *p = 0.034* | β = -0.15,  t = -1.17,  p = 0.125 | β = -0.18,  t = -1.47,  p = 0.074 | β = -0.03,  t = -0.25,  p = 0.403 | β = -0.19,  t = -1.51,  p = 0.069 | β = -0.11,  t = -0.77,  p = 0.222 |
| Braak V/VI Cohort (N = 25, df = 17/18) | | | | | | |
| Tangles (Q) | β = -0.30,  t = -1.40,  p = 0.089 | *β = -0.41,*  *t = -2.32,*  *p = 0.016* | β = -0.33,  t = -1.62,  p = 0.061 | β = -0.10,  t = -0.48,  p = 0.317 | β = -0.15,  t = -0.77,  p = 0.227 | β = 0.09,  t = 0.41,  p = 0.655 |
| Threads (Q) | β = 0.28,  t = 1.21,  p = 0.879 | β = 0.19,  t = 1.01,  p = 0.837 | β = -0.17,  t = -0.86,  p = 0.202 | β = 0.31,  t = 1.53,  p = 0.929 | β = -0.30,  t = -1.51,  p = 0.074 | β = 0.04,  t = 0.18,  p = 0.569 |
| MTL pTDP-43 (SQ) | β = 0.10,  t = 0.39,  p = 0.648 | β = 0.01,  t = 0.03,  p = 0.512 | β = -0.04,  t = -0.19,  p = 0.427 | β = 0.05,  t = 0.22,  p = 0.584 | β = -0.09,  t = -0.41,  p = 0.345 | β = 0.03,  t = 0.11,  p = 0.544 |

**Supplementary Table 9: Pathology to structure analyses in cases with quantitative p-tau pathology measures and ipsilateral MRI within 3 years prior to death.** The standardized β coefficient, t statistic, and p value for the association of each pathology measure with each imaging ROI is given within each cohort (top: whole cohort, N = 51, bottom: subset of cases with Braak Stage V/VI, N = 25). This analysis could not be repeated in the LATE-NC group due to small sample size (N = 7). Degrees of freedom (df) are also given for each cohort; the first number is for AH/PH associations due to the additional covariate of ICV, while the second number is for ERC, BA35, BA36, and PHC associations. Associations that would survive Bonferroni correction (P < 8.33e-03) are bolded; associations that are significant at P < 0.05 but would not survive corrections are italicized. All models were covaried for age, MRI field strength, and sex, but not antemortem interval; models for AH and PH also included ICV as a covariate. AH (vol) = Anterior hippocampus volume, PH (vol) = posterior hippocampus volume, ERC (thk) = entorhinal cortex median thickness, BA35 (thk) = Brodmann area 35 median thickness, BA36 (thk) = Brodmann area 36 median thickness, PHC (thk) = parahippocampal cortex median thickness. Tangles (Q) = quantitative tangles summary measure, Threads (Q) = quantitative threads summary measure, MTL pTDP-43 = semi-quantitative MTL pTDP43 rating

**Supplementary Fig.1 Comparison of aggregation methods for generating quantitative p-tau pathology measures.** An expert rater (SA) rated 64 sampling regions for tangle and thread pathology from 0-3 by a visual read. Quantitative measurements for each sampling region were generated by aggregating activation measures from segments within the sampling region. Aggregation methods include taking the mean, median, and maximum activation values, as well as various percentiles (25th, 75th, 90th, 95th, and 99th); each panel shows the quantitative measurements in each sampling region using one of these methods, grouped by the semi-quantitative expert rating of that pathology

** Supplementary Fig.2 Comparison of aggregation methods for generating quantitative pTDP-43 pathology measures.** An expert rater (JR) rated 93 sampling regions for neuronal/glial and neuritic pTDP-43 pathology from 0-3 by a visual read. Quantitative measurements for each sampling region were generated by aggregating activation measures from segments within the sampling region. Aggregation methods include taking the mean, median, and maximum activation values, as well as various percentiles (25th, 75th, 90th, 95th, and 99th); each panel shows the quantitative measurements in each sampling region using one of these methods, grouped by the semi-quantitative expert rating of that pathology

**Supplementary Fig.3 Average pathology measurements in each anatomical region of interest and the summary measure grouped by Braak stages**. Each column evaluates a specific inclusion type (A: tangles, B: threads, C: neuronal/glial pTDP-43, D: neuritic pTDP-43) in each pathological ROI stratified by Braak stages. The last row in each column shows the average summary measure for that pathology before normalization to the MTL p-tau/pTDP-43 rating such that all measures for a given inclusion are on the same scale. These plots contain data from all autopsies with quantitative p-tau data for tangles and threads (N = 196) or quantitative pTDP-43 data for neuronal/glial and neuritic pTDP-43 (N = 190). The number of cases within each pathological ROI varies, as not every region was annotated in every case due to tearing or mis-staining. Note that the DG-H measure for threads was not analyzed due to inaccurate classifying of mossy fibers in the dentate gyrus as threads. Error bars represent the standard error of the mean. CA = cornu ammonis, SUB/PrS = subiculum / presubiculum, CA1-SUB = cornu ammonis 1 / subiculum, DG-H = hilus of the dentate gyrus, DG-GCL = granular cell layer of the dentate gyrus, EC-med = medial portion of the entorhinal cortex visible on the section, EC-ctr = central portion of entorhinal cortex visible on the section, EC-lat = lateral portion of entorhinal cortex visible on the section, BA = Brodmann area

**Supplementary Fig.4 Average pathology measurements in each anatomical region of interest and the summary measure grouped by LATE stages**. Each column evaluates a specific inclusion type (A: tangles, B: threads, C: neuronal/glial pTDP-43, D: neuritic pTDP-43) in each pathological ROI stratified by LATE stages. The last row in each column shows the average summary measure for that pathology before normalization to the MTL p-tau/pTDP-43 rating such that all measures for a given inclusion are on the same scale. These plots contain data from all autopsies with quantitative p-tau data for tangles and threads (N = 196) or quantitative pTDP-43 data for neuronal and neuritic pTDP-43 (N = 190). The number of cases within each pathological ROI may vary, as not every region was annotated in every case due to tearing or mis-staining. Note that the DG-H measure for threads was not analyzed due to inaccurate classifying of mossy fibers in the dentate gyrus as threads. Error bars represent the standard error of the mean. CA = cornu ammonis, SUB/PrS = subiculum / presubiculum, CA1-SUB = cornu ammonis 1 / subiculum, DG-H = hilus of the dentate gyrus, DG-GCL = granular cell layer of the dentate gyrus, EC-med = medial portion of the entorhinal cortex visible on the section, EC-ctr = central portion of entorhinal cortex visible on the section, EC-lat = lateral portion of entorhinal cortex visible on the section, BA = Brodmann area

**Supplemental Fig.5 Scatterplots of pathology measures against semi-quantitative ratings of neuronal loss in the MTL.** Quantitative and semi-quantitative measures of each pathology were plotted against the average semi-quantitative rating of neuronal loss in the MTL. 196 cases were included in the p-tau analyses and 190 in the pTDP-43 analyses. Spearman correlations between each pathology measure and the MTL neuronal loss rating are shown in the top left of each plot. Tangles (Q) = quantitative tangles summary measure, Threads (Q) = quantitative threads summary measure, Neuronal/Glial pTDP-43 (Q) = quantitative neuronal/glial pTDP-43 summary measure, Neuritic pTDP-43 (Q) = quantitative neuritic pTDP-43 summary measure, MTL pTDP-43 = semi-quantitative MTL pTDP43 rating, MTL p-Tau (SQ) = semi-quantitative MTL p-Tau rating

**Supplemental Fig.6 Maps showing the association between pointwise cortical thickness and pathology measures in the 126 cases with both quantitative pathology measures and ipsilateral imaging**. Each row represents a different linear model (top: quantitative p-tau and pTDP-43 parameters used; middle: quantitative p-tau and semi-quantitative pTDP-43 measures used, as in the main text; bottom: semi-quantitative p-tau and pTDP-43 measures used). All models were covaried for age, antemortem interval, MRI field strength, and sex. The clusters outlined in black indicate regions where a significant association was found (p < 0.05), and the p-values are indicated with a line. To the right, a 3-D representation of the MTL with the subregions labeled is shown; the white matter section was not analyzed. AH = Anterior hippocampus, PH = posterior hippocampus, ERC = entorhinal cortex, BA35 = Brodmann area 35, BA36 = Brodmann area 36, PHC = parahippocampal cortex. WM = white matter. Tangles (Q) = quantitative tangles summary measure, Threads (Q) = quantitative threads summary measure, Neuronal/Glial pTDP-43 (Q) = quantitative neuronal/glial pTDP-43 summary measure, Neuritic pTDP-43 (Q) = quantitative neuritic pTDP-43 summary measure, MTL pTDP-43 = semi-quantitative MTL pTDP43 rating, MTL p-Tau (SQ) = semi-quantitative MTL p-Tau rating

1. Karypis G, Kumar V (1998) A Fast and High Quality Multilevel Scheme for Partitioning Irregular Graphs. SIAM J Sci Comput 20:359–392. doi: 10.1137/S1064827595287997
